# Deglacial stratification of the polar Southern Ocean

**DOI:** 10.1073/pnas.2502076123

**Published:** 2026-02-02

**Authors:** François Fripiat, Daniel M. Sigman, Xuyuan E. Ai, Cédric Dumoulin, Simone Moretti, Anja S. Studer, Bernhard Diekmann, Oliver Esper, Thomas Frederichs, Frank Lamy, Ling Liu, Frank Pattyn, Mareike Schmitt, Ralf Tiedemann, Gerald H. Haug, Alfredo Martínez-García

**Affiliations:** ^a^Department of Geosciences, Environment and Society, Université Libre de Bruxelles, Brussels 1050, Belgium; ^b^Climate Geochemistry Department, Max Planck Institute for Chemistry, Mainz 55128, Germany; ^c^Department of Geosciences, Princeton University, Princeton, NJ 08544; ^d^Department of Environmental Sciences, University of Basel, Basel 4056, Switzerland; ^e^Alfred Wegener Institute, Helmholtz Centre for Polar and Marine Research, Bremerhaven 27570, Germany; ^f^Center for Marine Environmental Sciences, University of Bremen, Bremen 28334, Germany; ^g^Department of Earth Sciences, ETH Zürich, Zürich 8092, Switzerland

**Keywords:** Southern Ocean, Antarctic Ice Sheet, deglaciation, stratification, N isotopes

## Abstract

It is widely accepted that meltwater from northern ice sheets drove changes in the Atlantic Meridional Overturning Circulation, impacting global climate. In contrast, data are lacking on the Antarctic Ice Sheet’s (AIS) effect on Southern Ocean overturning, despite the Southern Ocean’s central role in ventilating the ocean interior. Here, we present diatom-bound nitrogen isotope evidence indicating that during deglaciations, as wind-driven upwelling in the open Antarctic Ocean increased, AIS melting strengthened density stratification in the polar Antarctic Ocean. This change coincides with reconstructions of meltwater discharge and resembles model simulations of global warming including AIS meltwater forcing. The transient input of freshwater and, thus, buoyancy weakened Southern Ocean’s “lower cell” that outcrops in the polar Antarctic Ocean even as open Antarctic Ocean’s “upper cell” strengthened. Given evidence that deep-ocean ventilation and CO_2_ release were ongoing early in deglaciation, our data suggest that the open Antarctic Ocean, and not solely the most polar Antarctic Ocean, can directly ventilate the deep ocean. During the last deglaciation, the strongest density stratification in the polar Antarctic Ocean occurred just before the Antarctic Cold Reversal, when southern hemisphere warming paused and the northern hemisphere warmed sharply (i.e., during the Bølling–Allerød period). We propose that when combined with stronger wind-driven upwelling, the freshwater-driven redirection of upwelled water away from deep water formation and toward the upper cell deepened the global pycnocline, working to restart North Atlantic deep water formation in the Bølling–Allerød period. If so, polar ocean freshening in both hemispheres drove the “bipolar seesaw” events of deglaciation.

## Significance

The effect of global warming on the circulation of the Southern Ocean is complicated by the potential for interactions between wind-driven upwelling and surface buoyancy fluxes. Here, we study changes in the Southern Ocean’s surface conditions during the last two deglaciations, when Earth warmed rapidly. We demonstrate that during each of these events, meltwater from the Antarctic Ice Sheet temporarily stratified the upper ocean near Antarctica. The findings point to specific mechanisms for the early rise in atmospheric CO_2_ concentrations during deglaciations and the climatic “seesaw” behavior between the hemispheres, highlighting the potential impacts of the Antarctic Ice Sheet on the ocean’s ability to absorb heat and carbon dioxide in a warming world.

**Fig. 1.**
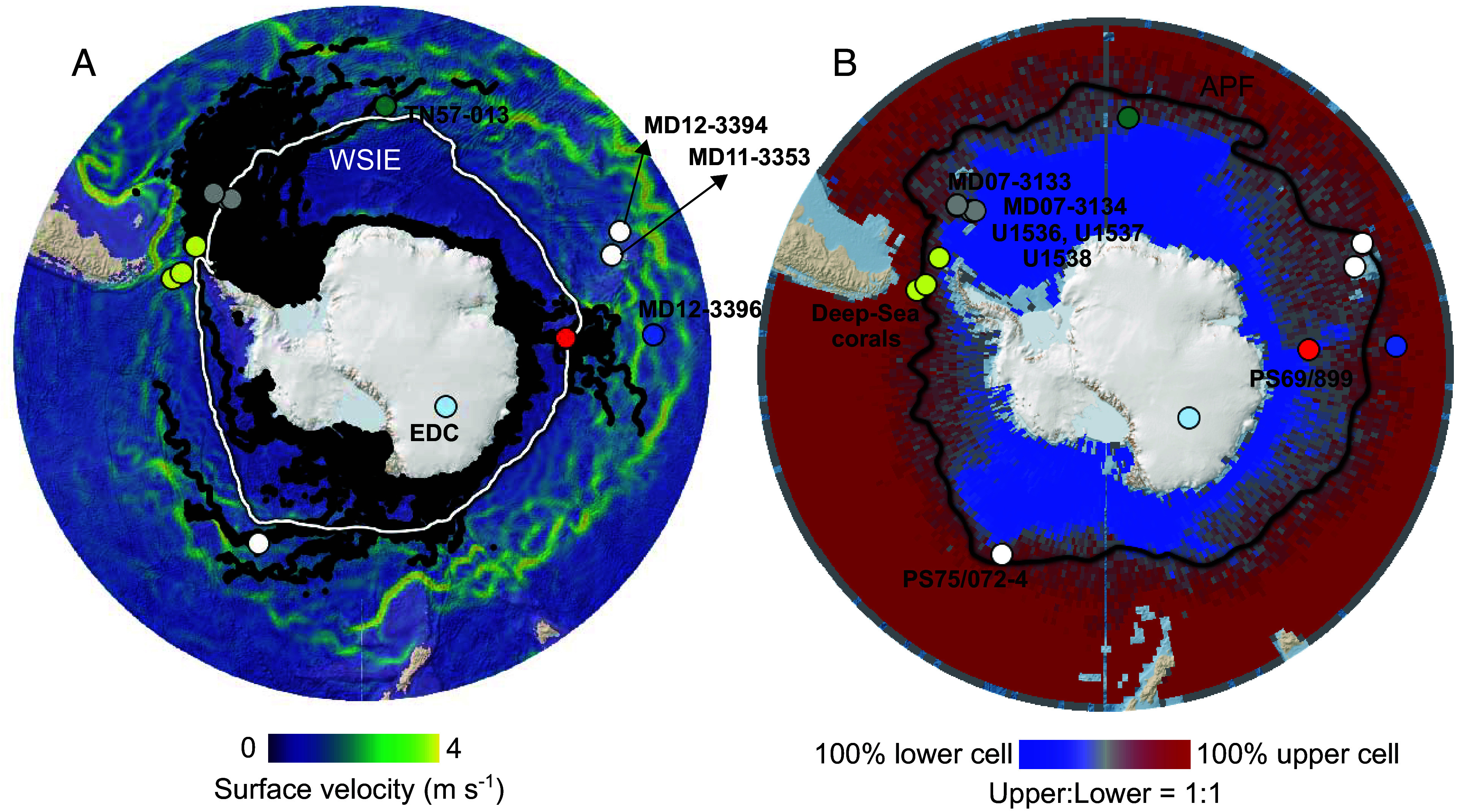
Maps of the Southern Ocean with the locations of sediment cores. The red circle shows the location of the diatom-bound δ^15^N record in the Polar Antarctic Zone (PS69/899-2; this study). (*A*) Surface current velocity (12) (color scale), Antarctic Iceberg Tracking database (13) (black tracks), and winter median sea-ice extent (14) (1981 to 2010; white line; WSIE). (*B*) Distinction between regions of the upper ocean feeding the upper and lower cells, respectively, based on particles released at 500 m depth in a high-resolution ocean model (15). The Antarctic Polar Front (APF) is shown as the black line (16). White circles indicate previously published diatom-bound δ^15^N records from the Open Antarctic Zone (17, 18) (PS75/072-4, MD11-3353, and MD12-3394) to which we compare our record from the Polar Antarctic Zone. The light-blue circle is the EPICA Dome C ice-core record (19–21) (EDC). Gray circles indicate sediment cores with reconstructions of the ice-rafted-debris flux (22–24) (MD07-3133, MD07-3134, U1536, U1537, and U1538). The petrol circle indicates a reconstruction since the Last Glacial Maximum of wind-driven upwelling near the APF based on the thorium-normalized opal accumulation rates (25) (TN57-013). The blue circle indicates a reconstruction of deep ocean O_2_ based on authigenic U levels (26) (MD12-3396). Yellow circles indicate a reconstruction of subglacial discharge inferred from deep-sea coral δ^234^U measurements (27).

In the Southern Ocean’s Antarctic Circumpolar Current (Fig. 1*A*), westerly winds force upwelling by causing a divergent meridional Ekman transport at the surface of the Antarctic Zone (AZ) south of the Antarctic Polar Front (APF). This process allows previously sequestered CO_2_ to be released back into the atmosphere (1) while also encouraging the ocean’s absorption of anthropogenic CO_2_ and of the excess atmospheric heat associated with global warming (2). Recent observations suggest that westerly winds are migrating poleward and intensifying (3), which appears to be increasing the ventilation of the upper AZ and global pycnocline (roughly the upper 1.2 km) (4). While potentially accelerating the exchange of CO_2_ and heat between the atmosphere and the interior of the ocean (2), the upwelling may also bring warm deep water, derived from Circumpolar Deep Water (CDW), onto the Antarctic continental shelf, where, along with atmospheric warming, it can accelerate the melting of the Antarctic Ice Sheet (AIS) (5). One possible consequence of the resulting meltwater addition to the ocean is the promotion of upper ocean density stratification, which may isolate the upwelled warm deep water from the surface (6). This mechanism may cool the sea surface (or slow its warming) while warming the shallow subsurface, which may further accelerate ice-sheet loss through basal melting of ice shelves (7, 8). While climate models simulate wind and buoyancy forcings, their relative importance and interactions in the Southern Ocean depend on poorly constrained aspects of the model, such as isopycnal and diapycnal mixing, and instrumental data are too limited for model validation (9–11). This situation adds to the uncertainty of models’ hindcasts of Southern Ocean conditions during past climates and their predictions of the future.

As a complementary window into the AIS’s impact on the upper ocean in the AZ and the relative contribution of different forcings (wind vs. buoyancy), we examine the Southern Ocean’s circulation over the last full glacial cycle, encompassing two deglaciations when Earth’s temperature and sea level rose by 6 °C and 120 m, respectively (28). We measured the ^15^N/^14^N ratio (hereafter referred to in delta notation, as δ^15^N vs. Air) of organic nitrogen encapsulated within diatom frustules (i.e., diatom-bound N) in deep-sea sediments. Diatom-bound δ^15^N provides a metric of the ratio of nitrate uptake to gross nitrate supply (i.e., the degree of nitrate consumption) in AZ surface waters (17, 18). The degree of nitrate consumption is expected to decrease with stronger wind-driven upwelling, which increases the gross supply of nitrate, unless this increase is met by a proportionally equivalent increase in nitrate assimilation by phytoplankton. Conversely, the degree of nitrate consumption will increase with stronger density stratification of the AZ upper water column. An increase in stratification would lower gross nitrate supply from below while also possibly improving the light conditions for phytoplankton growth, leading to more complete consumption of the available nitrate pool.

## Reconstructions of Surface Conditions in the Polar Southern Ocean.

**Fig. 2.**
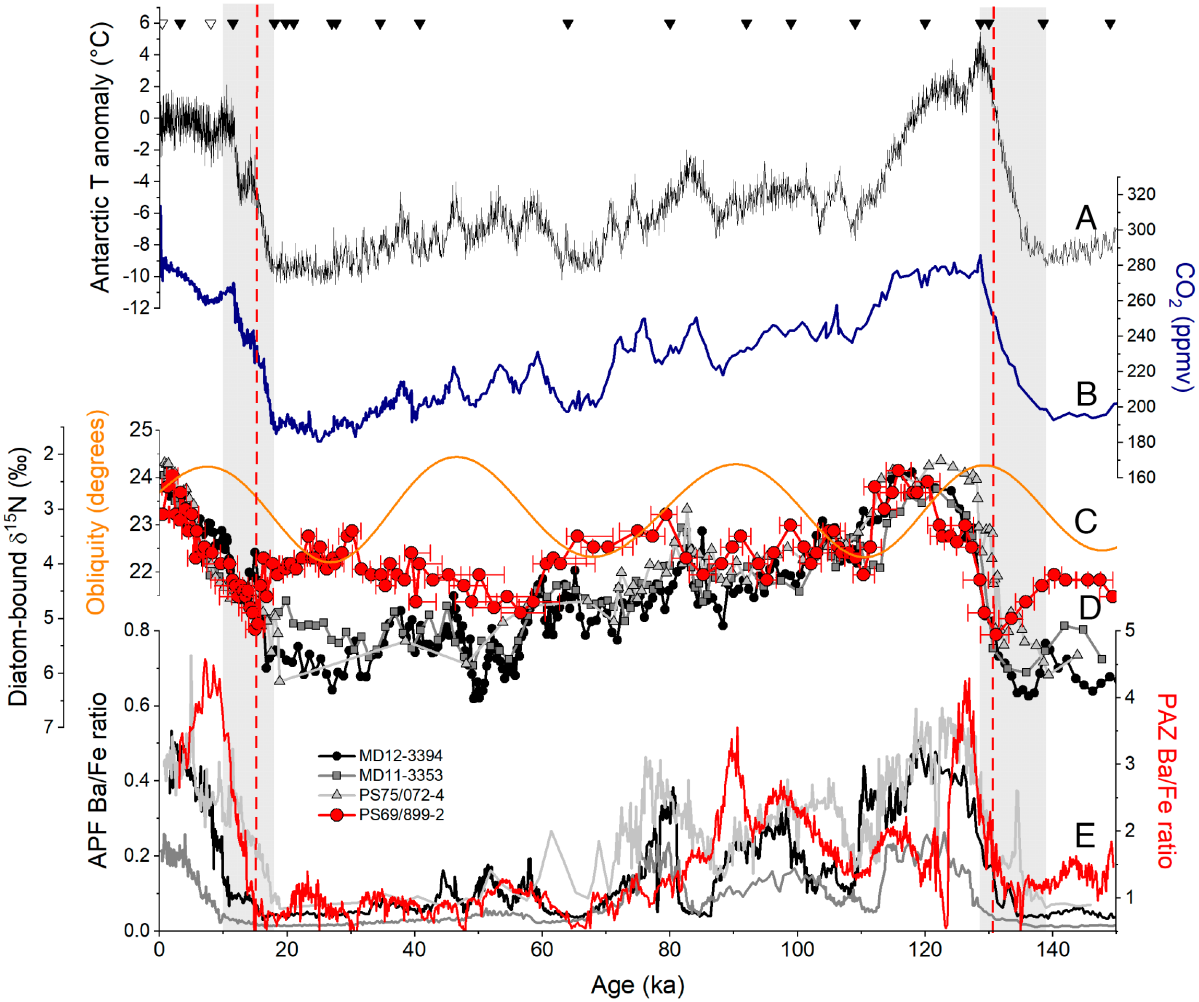
Ice-core and sedimentary records of Antarctic climate, atmospheric CO_2_, surface nitrate consumption, and export production in the Southern Ocean over the last glacial cycle. (*A*) Reconstruction of air temperature anomaly in Antarctica (20) (black). (*B*) Atmospheric CO_2_ reconstruction (21) (dark blue). (*C*) Earth’s obliquity (i.e., axial tilt) (orange) (37). (*D*) Diatom-bound δ^15^N records near the Antarctic Polar Front (APF), with black circles for MD12-3394 in the Indian Sector (18), dark-gray squares for MD11-3353 in the Indian Sector (18), light-gray triangles for PS75/072-4 in the Pacific Sector (17), and the diatom-bound δ^15^N record of PS69/899-2 in the Polar Antarctic Zone (PAZ; this study; filled red circles). Note inverse orientation of the vertical axis. Red error bars in (*D*) represent the 68.2% CI of the diatom-bound δ^15^N record of PS69/899-2 for age uncertainties. (*E*) From the same sediment cores, Ba-to-Fe ratio, a proxy for export production (30), with the colors corresponding to those of the diatom-bound δ^15^N records in (*D*). Gray shaded areas indicate the deglaciations. The dashed vertical red lines highlight the transient deglacial declines in the gross supply of nitrate to PAZ surface waters. The age model tie-points for PS69/899-2 are indicated with triangles along the top (filled symbols for the piston core and open symbols for the trigger core).

**Fig. 3.**
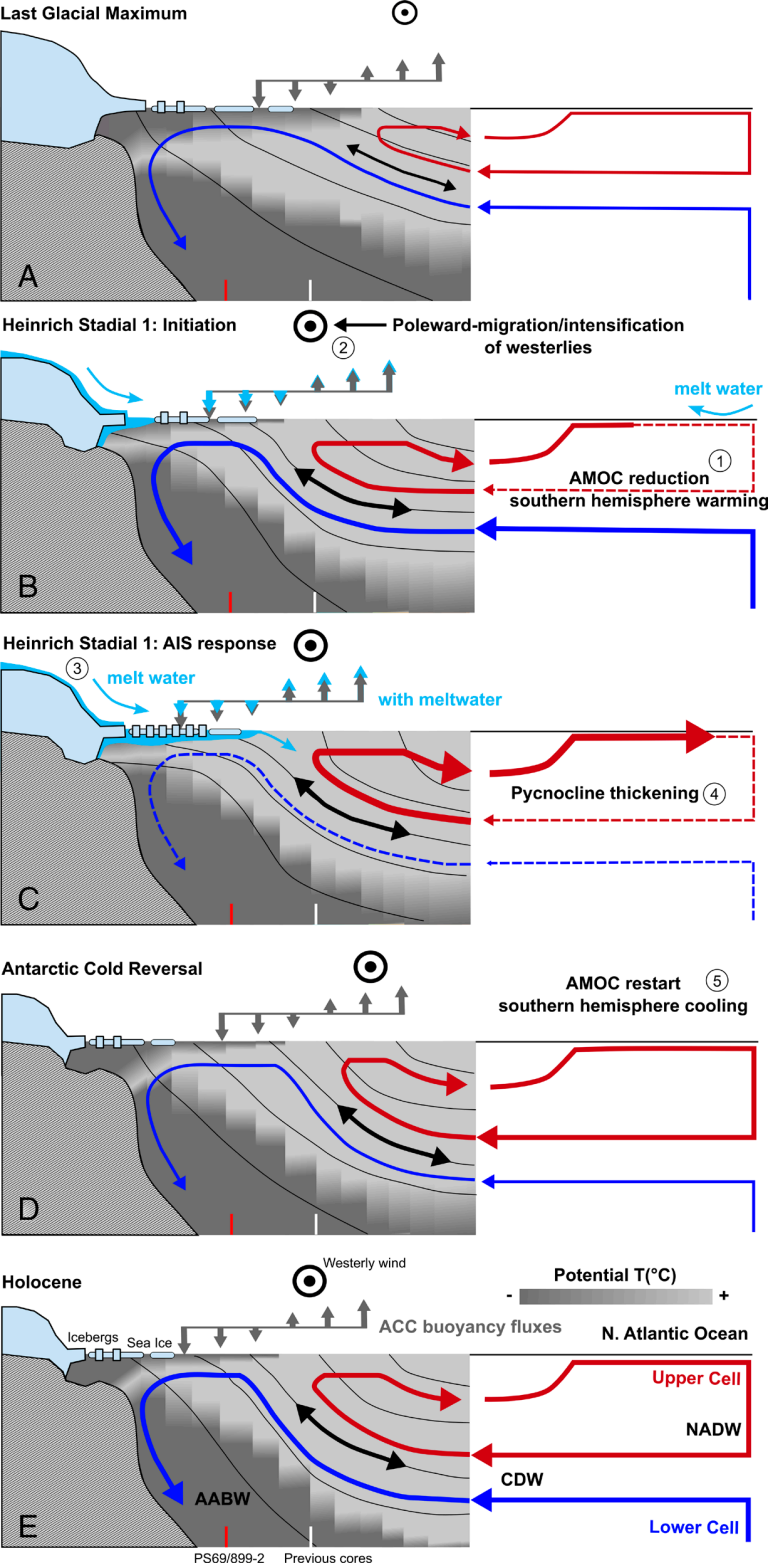
The proposed Southern Ocean’s overturning circulation since the Last Glacial Maximum and teleconnections with the North Atlantic Ocean. (*A*) the Last Glacial Maximum, (*B*) the initiation of the Heinrich Stadial 1, (*C*) Antarctic Ice Sheet response during Heinrich Stadial 1, (*D*) the Antarctic Cold Reversal, and (*E*) the Holocene. The numbers in (*B, C,* and *D*) indicate the sequence of events. Thin black lines represent isopycnals. The double-direction arrow indicates isopycnal mixing, which is sensitive to Ekman upwelling (38) and is proposed here to provide a mechanism for deep ocean ventilation and CO_2_ release, independent of the advection associated with the lower cell. Line thickness for the arrows denotes a change in flow rate, with thin dashed lines representing the lowest rate. The grayscale in *Left* panels indicates potential temperature, with darker being colder. The representation of meltwater discharge in panels *B* and *C* is intended to graphically highlight its impact on Southern Ocean dynamics, rather than to depict the vertical extent of the input. Gray arrows indicate the meridional buoyancy gradient at the Antarctic Circumpolar Current (ACC) surface, while cyan arrows in panels *B* and *C* show the modifications on the meridional buoyancy gradient induced by meltwater discharge. The right side of the figure shows the proposed interactions with the Meridional Overturning Circulation and global pycnocline thickness. The colors for the lower and upper cells correspond to those in Fig. 1*B*. AIS refers to the Antarctic Ice Sheet, AABW to Antarctic Bottom Water, CDW to Circumpolar Deep Water, and NADW to North Atlantic Deep Water. The vertical lines schematically illustrate the locations of core PS69/899-2 and previously investigated cores.

Previous studies in the AZ indicate higher nitrate consumption in surface waters during ice ages, as recorded by higher diatom-bound δ^15^N (17, 18) (Fig. 2*D*). In addition, proxy data such as lower biogenic barium and opal fluxes point to a concurrent decrease in export production (i.e., reduced sinking of organic matter out of the surface ocean) (29, 30) (Fig. 2*E*). Here, the sedimentary barium-to-iron (Ba/Fe) ratio is taken as a proxy for export production, as Ba/Fe records have aligned with thorium-normalized opal flux measurements when obtained from the same core (17, 30). Higher nutrient consumption and lower export production, together, suggest a reduction in gross nitrate supply to the surface, due to a decrease in the exchange of water between the surface and underlying ocean interior during ice ages (31). This decrease in upper AZ exchange is best explained by a decrease in wind-driven upwelling, caused by equatorward migration and weakening of the westerly winds in response to global cooling (18, 31–33) (Fig. 3*A*). Alternative mechanisms include an expansion of the region of buoyancy loss (34, 35) and the reduced sensitivity of seawater density to temperature at low temperatures (36).

However, these diatom-bound δ^15^N records are confined to the so-called “opal belt” near the APF (Fig. 1). This area is located in the “upper cell” of the Southern Ocean, which ventilates the global pycnocline and ultimately feeds North Atlantic Deep Water (NADW) formation (15) (Fig. 3), and it is far from the AIS. In the polar Antarctic Zone (PAZ), surface waters have a greater tendency to return to the deep ocean, for example, with the formation of Antarctic Bottom Water (AABW) near Antarctica, as part of the “lower cell” of Southern Ocean overturning (15) (Figs. 1 and 3). The low concentration of opal in PAZ sediments, coupled with challenges in establishing reliable chronologies due to limited biogenic carbonate, has hitherto hindered diatom-bound δ^15^N reconstructions, despite the critical role of this region in ventilating the ocean interior.

To extend glacial-interglacial diatom-bound δ^15^N data further south into the PAZ, we report measurements from sediment core PS69/899-2 in the Indian sector at the northern margin of the seasonal sea-ice zone (Fig. 1; 59.62°S, 85.67°E, water depth = 4,128 m; see methods) (39, 40). The core is situated near the present-day winter sea-ice edge (Fig. 1*A*) and along one of the primary routes for icebergs from Antarctica to the Southern Ocean (13). Sea-ice reconstructions suggest that, during the Last Glacial Maximum (LGM), the core site was in the seasonal sea-ice zone (41). This record covers the last full glacial cycle (back to 150 ka), encompassing two deglaciations. The age-depth model uses a combination of stratigraphic markers (42), with down-core age uncertainties estimated with the *UNDATABLE* Matlab routine (*SI Appendix*, section S1) (43).

As in sediment records near the APF, PAZ diatom-bound δ^15^N is on average higher in the glacial intervals of core PS69/899-2, supporting a greater degree of nitrate consumption during the ice ages (Fig. 2*D*). This, in conjunction with decreased export production (Fig. 2*E*), indicates reduced exchange between surface water and the underlying ocean relative to the interglacials. The shared PAZ and APF diatom-bound δ^15^N rise from the last interglacial into the early glacial inception (i.e., a 1.5‰ rise in δ^15^N from 120 to 110 ka) calls for a common mechanism of change (Fig. 2*D*). Previous work on the APF records has pointed to a reduction of the Southern Ocean’s upper cell, with an equatorward shift and weakening of the westerlies reducing Ekman transport and associated wind-driven upwelling (17, 18). This reduction in Ekman transport would have been compensated by the opposing response of eddies (44), but only partially (45, 46), such that the “residual” (net large-scale) transport of the upper cell and thus nitrate supply to the AZ surface would have decreased. In addition to large-scale advective flow, isopycnal mixing with the ocean interior also supplies nutrients to the AZ surface. Eddy-resolving numerical models suggest that isopycnal mixing along the upwelling limb of the upper cell depends on wind strength (38). Thus, the weakening of the westerlies may have further reduced the gross nitrate supply by decreasing isopycnal mixing.

The decline in upwelling would have increased the water residence time in the upper AZ, which may have allowed the halocline to strengthen and further limit the exchange of water between the surface and the deep ocean (32), potentially explaining part or all of the PAZ δ^15^N rise. Halocline strengthening in the PAZ may also have restricted the processes that ventilate the deep ocean, which include the formation of AABW near the coast of Antarctica, open ocean deep convection in offshore polynyas, and widespread diffuse vertical mixing across the base of the winter mixed layer (38, 47) (Fig. 3*A*). Even if the PAZ surface continued to serve as an important source for the deep ocean, our results indicate that it did so at reduced rates, consistent with radiocarbon data from the LGM deep ocean (48, 49), and with less unused nitrate being conveyed into the interior.

From the glacial inception to the LGM (i.e., from ~110 to ~20 ka), PAZ diatom-bound δ^15^N lacks a clear secular trend, instead showing a moderate correlation with Earth’s obliquity (Pearson correlation = 0.38, *P*-value of <0.01; Fig. 2 *C* and *D*). This contrasts with diatom-bound δ^15^N records near the APF, which show an additional δ^15^N rise of ~1.5‰ around 60 to 54 ka. Near the APF, the continued rise in diatom-bound δ^15^N suggests a progressive increase in the degree of nitrate consumption over the course of the ice age. This could be explained by 1) a further decrease in the supply of subsurface nutrients to the surface ocean near the APF, as suggested by a progressive reduction in export production (17, 18), 2) an increase of the nitrate drawdown associated with iron fertilization in the Subantarctic Zone to the north (50), the signal of which may have been mixed southward into the AZ near the APF, and/or 3) “slumping” of isopycnals (31) that homogenized surface conditions between the Subantarctic Zone and the open AZ. Some persistent rate of surface-subsurface exchange, associated with the lower cell and/or a background of wintertime vertical mixing, may have buffered the PAZ against further reductions in the nitrate supply. Alternatively, stronger constraints on phytoplankton growth in the PAZ, such as from light, may have led to a stable degree of nitrate consumption despite a progressive decline in nitrate supply.

**Fig. 4.**
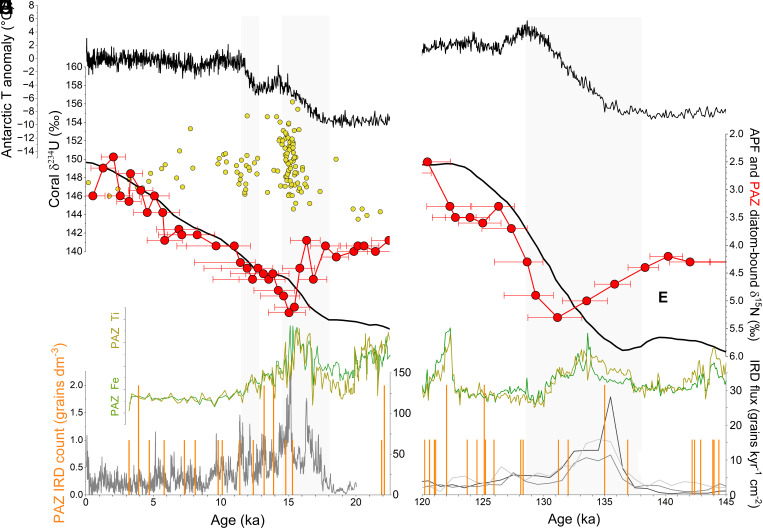
Changes in the Southern Ocean and Antarctic Ice Sheet over the last (*Left*) and penultimate (*Right*) deglaciations. (*A* and *B*) Air temperature anomaly in Antarctica (20) (black). (*C*) Subglacial discharge inferred from deep-sea coral δ^234^U measurements (yellow circles), where higher δ^234^U values indicate increased subglacial discharge (27). (*D* and *E*) Surface nitrate consumption based on diatom-bound δ^15^N of PS69/899-2 in the Polar Antarctic Zone (PAZ; red) and a diatom-bound δ^15^N stack for the records (17, 18) near the Antarctic Polar Front (APF; black). Error bars in (*D*) represent the 68.2% CI of the diatom-bound δ^15^N record of PS69/899-2 for age uncertainties. (*F* and *G*) XRF Ti and Fe concentrations for PS69/899-2. (*H* and *I*) IRD flux for the last (23) and penultimate (22) deglaciations (gray line), and IRD counts (orange) for PS69/899-2.

**Fig. 5.**
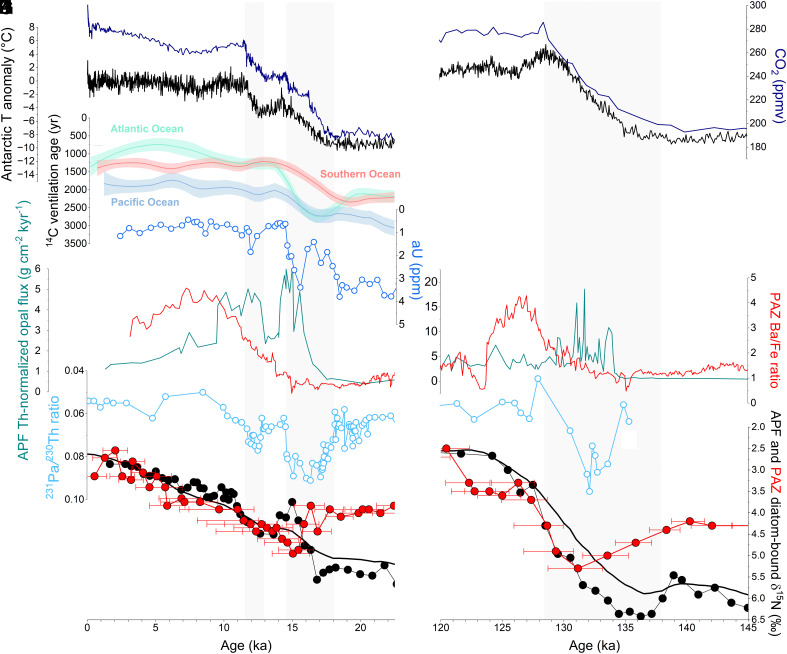
Changes in the Southern Ocean ventilation and atmosphere over the last (*Left*) and penultimate (*Right*) deglaciations. (*A* and *B*) Atmospheric CO_2_ reconstruction (21) (dark blue). (*C* and *D*) Air temperature anomaly in Antarctica (20) (black). (*E*) ^14^C ventilation ages for the deep Atlantic, Southern, and Pacific Ocean (49). (*F*) Deep ocean O_2_ concentration based on authigenic U (aU) of MD12-3396 (26) (blue). (*G* and *H*) Export production based on XRF Ba-to-Fe ratio of PS69/899-2 (red) and thorium-normalized opal flux of TN57-13 (petrol) (25, 30), in the Polar Antarctic Zone (PAZ) and in the Antarctic Polar Front (APF), respectively. (*I* and *J*) Strength of the Atlantic Meridional Overturning Circulation (cyan) based on ^231^Pa-to-^230^Th ratio in North Atlantic sediments (52, 53). (*K* and *L*) Surface nitrate consumption based on diatom-bound δ^15^N of PS69/899-2 in the PAZ (red) and MD12-3394 (18) near the APF (black). Error bars in (*D*) represent the 68.2% CI of the diatom-bound δ^15^N record of PS69/899-2 for age uncertainties.

Remarkably, in contrast to the APF records that show a deglacial decrease in δ^15^N, our new PAZ record indicates a transient rise in diatom-bound δ^15^N during each of the last two deglaciations (Fig. 2*D*, dashed red lines). This finding is further supported by a direct comparison of ${\mathrm{TEX}}_{86}^{L}$-based sea surface temperature and diatom-bound δ^15^N values plotted against depth in core PS69/899-2 (51) (*SI Appendix*, Fig. S7). The comparison indicates that these transient δ^15^N maxima occurred during deglaciations, irrespective of the age-depth model. At these times, PAZ diatom-bound δ^15^N not only shifts toward open AZ δ^15^N values but also rises above it for a brief period (Figs. 4 and 5). Since these δ^15^N rises were not accompanied by a peak in productivity (Fig. 2*E*), the data imply a transient decline in the gross supply of nitrate into the surface waters. We interpret this transient feature during the deglaciation as a response of the upper PAZ to the melting of the AIS, which induced a meltwater discharge that was apparently sufficient to stratify the upper ocean near Antarctica and reduce the strength of the Southern Ocean’s lower cell (Fig. 3).

## Antarctic Ice Sheet, Meltwater Discharge, and Stratification of the Polar Southern Ocean.

While the AIS contracted after the LGM, its deglacial history is uncertain due to sparse and unevenly distributed geological and glaciological observations (54). However, records of Ice Rafted Debris (IRD) accumulation from Iceberg Alley in the Atlantic sector (Fig. 1*A*) (22, 23) support a rise in AIS mass loss and meltwater discharge during deglaciations, coinciding with the transient rises in δ^15^N in core PS69/899-2 (Fig. 4). During the penultimate deglaciation, the δ^15^N maximum appears to lag behind the IRD flux peak, though this may reflect uncertainties in our age model. For the last deglaciation, the δ^15^N rise coincides with evidence of subglacial discharge through the Drake Passage (27) (Fig. 4*C*) and widespread AIS retreat, as supported by some observational and modeling studies (54, 55).

The link between the deglacial increase in diatom-bound δ^15^N and ice sheet discharge is further supported by concurrent peaks in terrigenous elements [e.g., titanium (Ti) and iron (Fe)] in core PS69/899-2 (Fig. 4 *F* and *G*), despite the significantly lower IRD presence compared to records closer to iceberg source regions (Fig. 4 *H* and *I*). This lower IRD abundance is expected, as most icebergs melt before reaching this remote site. Glacial-interglacial variations in terrigenous components align with ice-core dust records, reflecting higher dust fluxes during ice ages (*SI Appendix*, Fig. S1) (19). However, the deglacial peaks in Ti and Fe are consistent with records of IRD rather than with ice-core dust records (Fig. 4) (19, 22, 23). Fine terrigenous particles generated by glacial erosion can be transported farther than larger grains, which explains the deglacial peaks in terrigenous constituents despite the near absence of visibly identifiable IRD. In any case, while a large IRD flux should signal grounded ice loss, a perfect correlation between ice loss and IRD flux is not expected (56).

Together, data suggest that a substantial meltwater flux from the AIS early in the deglaciations caused the surface water near Antarctica to become fresher, and thus more buoyant, increasing the density stratification of the PAZ upper ocean (Fig. 3*C*). Because the overturning circulation must cross isopycnals in the mixed layer to close the circulation, this buoyancy input would have weakened the lower overturning cell (34, 57). The reduction in upper ocean vertical mixing and the weakening of the lower cell caused a transient reduction in the gross nitrate supply to the PAZ surface (Fig. 3*C*). Even if meltwater is discharged into specific sectors of the AZ, coastal and offshore currents will transport it around Antarctica within years to decades (9, 58).

Present-day mass loss of the AIS occurs by basal melting of floating ice shelves and iceberg calving (59, 60). Basal melting is closely tied to oceanic heat supply into sub-ice shelf cavities, which is accelerated where and when relatively warm CDW intrudes onto the continental shelf. The westerlies and wind-driven upwelling offshore in the Southern Ocean shoal CDW in the PAZ, facilitating its flow onto the shelf (5). As a result, a relationship between wind forcing and AIS mass loss is both expected and observed in the context of present-day global warming (60, 61). For example, warm-water sub-ice shelf cavities, where most of the basal melting occurs today, are observed where the Antarctic Circumpolar Current is situated near the continental shelf break (62).

As with present-day global warming (3), the westerlies are thought to have migrated poleward and intensified during deglaciations (32) (Fig. 3*B*), although the proxy support for this is debated (33, 63). Opal burial rates in AZ sediments have led to the inference of two intervals of enhanced upwelling during the last deglaciation, which coincided with Antarctic warming (25) (Fig. 5 *C* and *G*). Diatom-bound δ^15^N records near the APF indicate that these two intervals were accompanied by increases in surface nitrate concentration, consistent with the upwelling interpretation (Fig. 5*K*). The first of these intervals corresponds to the transient rise in PAZ diatom-bound δ^15^N and the first deglacial peak in IRD flux from Iceberg Alley. This initial interval of enhanced upwelling, likely from a poleward migration of the westerlies, may have triggered melting of the AIS along bathymetric troughs by increasing oceanic heat supply to sub-ice shelf cavities (Fig. 3*B*). At the same time, meltwater inputs may have prevented the emergence of these interior isopycnals, redirecting their heat transport toward the ice shelves rather than the ocean surface (64). While the glacial deep ocean was substantially colder than today, CDW is modified by mixing with the overlying low-latitude pycnocline (65), which would likely have maintained an adequately high temperature to drive melting in the sub-ice shelf cavities, even under glacial and deglacial conditions. While freshening around Antarctica reduced buoyancy flux and weakened the lower overturning cell, the northward advection of meltwater into the open AZ enhanced buoyancy flux there, which should have further strengthened the upper overturning cell (34, 57). Thus, the meltwater-associated buoyancy input to the Southern Ocean may have shifted the balance between the Southern Ocean’s lower and upper cells, weakening the lower cell while strengthening the upper cell (Fig. 3).

Buoyancy fluxes are central to the residual circulation of the Southern Ocean (34, 57), and their consideration has engendered debate as to whether wind stress can play a primary role in setting the overturning (45, 46). One view in support of winds as a driver of the overturning is that the westerly winds cause Ekman transport of open AZ surface waters toward lower latitudes, where these waters become more buoyant due to air–sea flux, thus satisfying the need for a buoyancy balance in the overturning circulation (45). Our data would appear to require a role for the winds in glacial/interglacial changes in the overturning. Specifically, the diatom-bound δ^15^N records indicate that the Antarctic meltwater discharge was a transient deglacial feature. Thus, the continued elevation of upwelling rates throughout the deglaciation and into the interglacial requires a nonmeltwater forcing, such as warming-associated southward-migration of the westerly winds. In summary, the accumulated data indicate that the open AZ and PAZ changes were initiated by wind-driven open AZ upwelling changes, which spurred meltwater release that weakened the Southern Ocean’s lower cell while further strengthening its upper cell.

The initial warming interval in Antarctica in the context of the last deglaciation corresponds with Heinrich Stadial 1 (HS1; ~18 to 14.7 ka), a period marked by a dramatic reduction in the Atlantic Meridional Overturning Circulation (AMOC) due to an influx of freshwater to the Nordic Seas, presumably from net loss of the northern hemisphere ice sheets (52, 53) (Fig. 5*I*). The weakening of the AMOC cooled the northern hemisphere while driving warming in the southern hemisphere (66) (Fig. 3*B*). While the impact of meltwater discharge on the AMOC is well documented, with a similar collapse/reduction during the penultimate deglaciation (Fig. 5*J*), the effect of Antarctic melting on the overturning circulation in the Southern Ocean has not received comparable attention. Our findings reveal that buoyancy forcing also impedes the overturning near Antarctica (lower cell in Fig. 3) during deglaciations (Fig. 5), even as poleward migration and intensification of the westerlies increased overturning rates near the APF (upper cell in Fig. 3 *B* and *C*). These coupled changes point to a “bipolar” interaction mechanism for deglacial changes in deep ocean ventilation.

## Bipolar Interaction in Deep Ocean Overturning.

During the last deglaciation, the strongest density stratification in the polar AZ occurred around 15 ka, just prior to the Antarctic Cold Reversal (ACR; ~14.7 to 13.0 ka). The ACR marked a pause in Antarctic deglacial warming trends (Fig. 4*A*) and coincided with the Bølling–Allerød warming in the Northern Hemisphere. As described above, in addition to reinforcing the density stratification in the PAZ, the spreading of meltwater from the AIS northward would add buoyancy to the open AZ, transforming CDW into lower-density surface waters that flow northward in the upper cell (Fig. 3*C*). Thus, the AIS melting would have caused upwelling CDW to be shifted from the lower cell to the upper cell. This would have synergized with the poleward migration and intensification of the westerlies in enhancing the upper cell of Southern Ocean overturning (Fig. 3*C*). Such a supercharging of the upper cell during HS1 may explain the exceptional peak in opal accumulation observed in the Atlantic sector of the AZ (25) (Fig. 5*G*).

The resulting increase in global pycnocline water formation from the Southern Ocean’s upper cell, simultaneous with a weakening in the lower cell, may have triggered the restart of the AMOC at the onset of the Bølling–Allerød period (67). The stronger upper cell would have deepened the pycnocline and increased sea-surface-height gradient between the tropical North Atlantic and the subpolar North Atlantic, encouraging high-latitude sinking of North Atlantic Deep Water (68). The temporary reduction in the lower cell and AABW formation may have further facilitated this process by allowing the density of the deep ocean to decrease, thus enabling dense surface waters from the North Atlantic to penetrate into the deep ocean (Fig. 3*D*).

Thus, the “seesaw” climate events of deglaciation may have been driven by polar ocean freshening in both hemispheres. To outline this bipolar mechanism, freshening of the North Atlantic at the onset of the deglaciation (HS1) would have cooled the Northern Hemisphere while warming the Southern Hemisphere. This warming intensified and shifted the southern westerlies poleward (18, 32, 33), driving melting of the AIS, which freshened the AZ. This, in turn, reinvigorated the AMOC, leading to Northern Hemisphere warming of the Bølling–Allerød period and the coincident Southern Hemisphere cooling of the ACR.

The positive correlation observed between diatom-bound δ^15^N and Earth’s obliquity during ice ages (Fig. 2 *C* and *D*) provides an additional sign of interaction between the AIS and the exchange of water and nutrients between the surface and deep ocean near Antarctica. When Earth’s obliquity increases, the rise in high-latitude insolation may cause an increase in Antarctic meltwater that, in turn, enhances buoyancy flux in the upper PAZ, leading to more complete nitrate consumption and, thus, higher δ^15^N. This process may be driven by insolation-induced surface ocean warming, which is subsequently transported into ice-shelf cavities along bathymetric troughs (69, 70). An alternative or possibly additional explanation is that higher obliquity weakens the westerly winds and their influence on Antarctic upwelling, which allows the PAZ halocline to strengthen (18). However, the obliquity signal in diatom-bound δ^15^N appears stronger in the PAZ than the open AZ, causing the PAZ and APF δ^15^N records to converge during high obliquity (Fig. 2*C*). This leads us to favor AIS meltwater discharge changes as the cause of the correlation between obliquity and PAZ diatom-bound δ^15^N.

## Deglacial Changes in Ocean Ventilation and Atmospheric CO_2_.

Marine radiocarbon data point to diminished deep ocean ventilation during ice ages. During the last deglaciation, there is evidence that reinvigoration of deep ventilation began early, i.e., during HS1 (48, 49), although it may have increased at subsequent times as well, continuing into the later deglaciation (67) (Fig. 5*E*). It appears that neither the North Atlantic nor the PAZ contributed to the early deep ocean ventilation, due to meltwater discharge in both regions (Fig. 3*C*). The early increase in deep Southern Ocean ventilation coincides with the initial deglacial rise in atmospheric CO_2_, suggesting that the cause of the early increase in deep ocean ventilation also allowed CO_2_ to escape from the deep ocean to the atmosphere (Fig. 5 *A* and *E*). With PAZ stratification and its inhibition of AABW formation, what can explain the observations of increased deep Southern Ocean ventilation and rising atmospheric CO_2_?

The traditional view of deep ocean ventilation focuses on the large-scale advective flows of NADW and AABW. However, the open AZ, closer to the APF, may be more important than previously assumed for the ventilation of the deep ocean. Eddy-resolving numerical models suggest that wind stress and its effects on isopycnal tilt and mesoscale eddies drive ventilation of the ocean interior through isopycnal mixing, with the isopycnals outcropping during the winter across much of the AZ (38). This mixing ventilates the deep ocean in the density range of the CDW that upwells to the surface as part of the upper and lower cells. Thus, despite stronger stratification in the PAZ near the Antarctic margin early in the deglaciation, intensification of the westerlies may have enhanced the ventilation of the deep ocean through the open AZ (Fig. 3, black double-direction arrow). This mechanism would tend to start its ventilation in the lower densities of the upwelling CDW and progressively ventilate higher-density waters (i.e., greater depths), consistent with some deglacial radiocarbon data (71–73).

However, evidence suggests that the polar AZ also contributed to the deglacial changes in deep ocean ventilation and atmospheric CO_2_. The heightened stratification in the upper PAZ around 15.5 ka likely increased the sequestration of CO_2_ in the deep ocean, countering the release of deeply sequestered CO_2_ in the open AZ and the waning of iron fertilization in the Subantarctic Zone (50) so as to explain the cessation of the rise in atmospheric CO_2_ during the latter portion of HS1 (Fig. 5 *A* and *K*). This interpretation is supported by a record of authigenic uranium from abyssal waters (3.6 km deep) in the Southern Ocean (26), which indicates a reversal of deep ocean oxygenation during late HS1, coinciding with the peak in PAZ stratification (Fig. 5 *F* and *K*). With the subsequent waning of PAZ stratification, acceleration of the lower overturning circulation cell (including AABW formation) could have then contributed to the increases in atmospheric CO_2_ in the later deglaciation and early Holocene (Fig. 3*C*). Indeed, this is likely required to offset the CO_2_-lowering effect of NADW invigoration at that time (52, 67) (Fig. 5i).

## Implications for the Future.

Our data indicate that the partial melting of the AIS early in deglaciations induced a transient rise in the buoyancy flux near Antarctica, weakening the lower cell of the overturning circulation and inducing a density stratification of the upper polar AZ. In the open AZ near the APF, previous records suggest a concurrent deglacial rise in upwelling rates, leading to a strengthening of the upper overturning circulation cell (17, 18). Both observations can be explained by a poleward migration and intensification of the westerlies and its effect on ocean circulation and melting at the AIS margin, which in turn reinforced the weakening of the lower cell and a strengthening of the upper cell. This set of deglacial changes is remarkably similar to recent observations across the Southern Ocean during the present-day global warming (9, 74). These present-day observations are not simulated in models unless the model forcing includes the increasing meltwater flux from the AIS, which has not been the case for IPCC CMIP simulations (6, 9, 10).

In the context of the orbitally paced ice age cycles, deglacial stratification of the polar AZ was transient (Fig. 4), with the stratifying effects of meltwater discharge eventually being overwhelmed by increased wind-driven upwelling due to the poleward migration and intensification of the westerlies (18, 25, 33). In the case of anthropogenic global warming, AIS melting and stratification of the polar AZ may be maintained into the foreseeable future. Accordingly, we must ask how impactful this stratification will be for deep ocean ventilation, which is required for energetic and chemical equilibration between the atmosphere and the voluminous ocean interior. Models cannot yet convincingly answer this question, given uncertainties in AIS behavior and the model resolution required to capture all modes of ocean ventilation (38, 75). Our data from the last deglaciation, when compared to records of deep ocean ventilation and atmospheric CO_2_, imply that the wind-driven dynamics of the open AZ will maintain deep ocean ventilation even in the face of polar AZ stratification by meltwater. If correct, the Southern Ocean’s “door” to the deep ocean will remain “propped open” (2) for the foreseeable future, allowing the Southern Ocean to continue to absorb anthropogenic CO_2_ and the excess of atmospheric heat associated with global warming. At the same time, the redirection of the water upwelled in the AZ away from the Southern Ocean’s lower overturning cell and into its upper cell may help to stabilize North Atlantic deep water formation in the face of surface freshening in the high latitude North Atlantic (76).

## Materials and Methods

Piston core PS69/899-2 was retrieved during the R.V. Polarstern expedition ANT-XXII/9 in 2007 from the Indian sector of the Southern Ocean (59.62°S, 85.67°E, water depth = 4,128 m) (Fig. 2). The 22.63 m long sediment core consists of an alternation of diatom ooze, diatom mud, and diatom-bearing mud (77). It was located on the slope of the southeast margin of the Kerguelen Plateau (Fig. 1), at the northern edge of the winter maximum sea-ice extent (median for 1981 to 2010) (14) and south of the southern ACC front (16). A trigger core for the piston core (PS69/899-2TC) was also retrieved and consisted of 0.30 m of diatom ooze.

Diatom-bound δ^15^N measurements (39, 40) were obtained from 5 to 10 g of sediments following the protocol of Studer et al. (17). First, diatoms were physically separated from the bulk sediment, then chemically cleaned. The physical separation of sediment samples was conducted by first dispersing them in a 2% Na-polyphosphate solution, which was sieved at 63 µm to remove the larger size fraction that can contain significant radiolarian shells and terrigenous material. Diatoms were then separated from the sediments using a series of steps, including i) eight to ten repeated differential settlings in 2% Na-polyphosphate solution to remove clays, ii) removal of calcium carbonate with 10% hydrochloric acid, and iii) a heavy-liquid density separation using Na-polytungstate solution (ρ = 2.15 g cm^−3^) and centrifugation to separate opal from remaining minerals. The last step was repeated until no sedimented fraction was deposited at the bottom of the centrifugation tube. We verified the separation of 60 samples with smear slides analyzed under the microscope during each step of the physical separation. Diatoms were then subjected to a three-step chemical cleaning, which began with a reductive step using a buffered sodium dithionite-citric acid solution at 80 °C in a water bath for 1 h to ensure the removal of oxide coatings. An oxidative treatment followed this step using 7% perchloric acid and then 72% perchloric acid in a boiling water bath for 1 and 2 h, respectively. The oxidative treatment ensured the elimination of external organic matter adhering to the diatom frustule surfaces. Cleaned diatom samples weighing 90 to 1,100 mg were rinsed with high-purity deionized water and dried in a clean oven at 60 °C.

Cleaned diatom samples were analyzed for their δ^15^N using the “persulfate-denitrifier” method (17, 78). 6 mg of clean opal was dissolved and the organic nitrogen released from the frustules oxidized to nitrate in a 1.0 to 1.5 M sodium hydroxide and 0.15 to 0.22 M potassium persulfate solution in an autoclave at 120 °C for 90 min. 20 nmol of nitrate was quantitatively converted to N_2_O gas by a strain of denitrifying bacteria (*Pseudomonas chlororaphis*, ATCC® 43928TM) that lacks an active N_2_O reductase enzyme (78). The isotopic composition of N_2_O was measured by gas chromatography/isotope ratio mass spectrometry using a custom-built N_2_O extraction and purification system online to a Thermo MAT 253 mass spectrometer (79). Measurements were referenced to air N_2_ using the nitrate reference materials IAEA-NO3, with a δ^15^N of 4.7‰, and USGS-34, with a δ^15^N of −1.8‰ (80). International reference organic standards (UGSG 40 and 41) and in-house internal diatom standards were processed along with the samples to ensure proper transformation of the N encapsulated in the diatom frustules into N_2_O. Replicate analyses of samples (n = 82) indicate a median 1 SD reproducibility of 0.08‰. Long-term reproducibility of 0.22‰ was determined on our in-house internal diatom standards measured twice in each batch, which underwent all steps from the physical separation of the bulk sediment to the diatom-bound δ^15^N analysis.

Diatom species composition (81) was analyzed from quantitative diatom slides prepared from core PS69/899-2, following the method in ref. 82 (*SI Appendix*, Fig. S2). For diatom counting, approximately 550 valves per sample were counted at 1,000× magnification using a Zeiss microscope, with identification at the species or species-group level. No correlations were found between diatom-bound δ^15^N and diatom species composition (*SI Appendix*, Fig. S3).

To determine the relative sedimentary elemental concentrations of Ba, Fe, and Ti, X-ray fluorescence scanning was performed using an Avaatech XRF core scanner. The data were collected at a 1 cm resolution for a duration of 30 s at 10 and 30 kV (77). Assuming that sedimentary Fe is of detrital origin, the Ba vs. Fe ratio provides an estimate of the sedimentary concentration of biogenic (or excess) Ba, used as a paleoproductivity proxy (30). This approach is further validated as this proxy is proportional with thorium-normalized opal fluxes if measured on the same cores (17, 30), despite large variability in paleoproductivity reconstructions between sediment cores (29). We use Fe and Ti as proxies for the supply of terrigenous constituents in PS68/899-2, which is further supported by their strong correlation with XRF Ca and K data (Pearson correlation coefficient >0.88; *P*-value of <0.01) along the record. During ice ages, higher concentrations of these constituents correlate with aeolian dust deposition in ice cores (*SI Appendix*, Fig. S1) (19), suggesting an aeolian origin. In contrast, deglacial peaks align with increases in IRD flux records (22–24), suggesting glacial erosion as the source for terrigenous constituents in PS68/899-2 (Fig. 4). Gravel clasts (>2 mm) were counted as ice-rafted debris (83) from 1 cm-thick X-radiographs of the sediment core (84, 85), although their abundance was significantly lower than in records from areas closer to iceberg source regions (22, 23).

The ${\mathrm{TEX}}_{86}^{L}$-paleothermometer is based on the temperature sensitivity of the relative abundance of isoprenoid glycerol dibiphantanyl glycerol tetraether lipids (GDGTs) with varying numbers of cyclopentane rings in their structure (86, 87). The temperature calibration proposed by Kim et al. (86) is used to estimate sea surface temperature (SST) in our study (51, 88). GDGTs were extracted from freeze-dried sediment samples using a solvent mixture of DCM:MeOH (1:1) on a Dionex Accelerated Solvent Extraction (ASE) 350 system with silica added inside the cell (89). GDGTs were then measured by high-performance liquid chromatography/mass spectrometry (HPLC-MS) using the method proposed by Hopmans et al. (90).

Paleomagnetic directions and magnetization intensities were measured on a superconducting rock magnetometer (2G Enterprises model 755 HR) on specimens of 6.2 cm^3^ volume sampled at 5 cm increments. Natural remanent magnetization (NRM) was measured on each sample before applying alternating field demagnetization using increments of 5 mT in fields up to 50 mT and 10 mT in fields up to 100 mT. Different types of laboratory-generated artificial remanences can be used to normalize NRM intensity for changes in the concentration of remanence carrying minerals and to estimate the relative paleointensity (RPI) of the Earth’s magnetic field as recorded in the sediments at the time of their deposition. Here, anhysteretic remanent magnetization (ARM) generated in a peak alternating field of 100 mT and a biasing DC field of 40 µT that was demagnetized at the same field steps as the NRM was used for normalization. RPI (91) was calculated using the so-called “slope-method” or pseudo-Thellier method (92, 93). RPI was computed as the slope of the regression line of NRM intensities plotted vs. the intensities of ARM_100mT_ for AF demagnetization levels 15 to 45 mT (NRM_demag15-45mT_/ARM_demag15-45mT_).

## Supplementary Material

Appendix 01 (PDF)

## Data Availability

Diatom-bound nitrogen isotope data, age–depth model, diatom species composition, TEX86-based sea surface temperatures, ice-rafted debris counts, and relative paleointensity data have been deposited in PANGAEA (39, 40, 42, 51, 81, 83, 88, 91).

## References

[1] I. Marinov, M. Follows, A. Gnanadesikan, J. L. Sarmiento, R. D. Slater, How does ocean biology affect atmospheric pCO_2_? Theory and models. J. Geophys. Res. **113**, C07032 (2008).

[2] J. L. Russell, K. W. Dixon, A. Gnanadesikan, R. J. Stouffer, J. R. Toggweiler, The southern hemisphere westerlies in a warming world: Propping open the door to the Deep Ocean. J. Clim. **19**, 6382–6390 (2006).

[3] N. C. Swart, J. C. Fyfe, Observed and simulated changes in the Southern Hemisphere surface westerly wind-stress. Geophys. Res. Lett. **39**, L16711 (2012).

[4] D. W. Waugh, F. Primeau, T. DeVries, M. Holzer, Recent changes in the ventilation of the Southern Oceans. Science **339**, 568–570 (2013). 23372011 10.1126/science.1225411

[5] D. M. Holland, K. W. Nicholls, A. Basinski, The Southern Ocean and its interaction with the Antarctic Ice Sheet. Science **367**, 1326–1330 (2020). 32193320 10.1126/science.aaz5491

[6] L. Menviel, A. Timmermann, O. E. Timm, A. Mouchet, Climate and biogeochemical response to a rapid melting of the West Antarctic Ice Sheet during interglacials and implications for future climate. Paleoceanography **25**, PA4231 (2010).

[7] C. J. Fogwill , Antarctic ice sheet discharge driven by atmosphere-ocean feedbacks at the last glacial termination. Sci. Rep. **7**, 39979 (2017). 28054598 10.1038/srep39979 PMC5215443

[8] A. Silvano , Freshening by glacial meltwater enhances melting of ice shelves and reduces formation of Antarctic Bottom Water. Sci. Adv. **4**, eaap9467 (2018). 29675467 10.1126/sciadv.aap9467 PMC5906079

[9] B. Bronselaer , Importance of wind and meltwater for observed chemical and physical changes in the Southern Ocean. Nat. Geosci. **13**, 35–42 (2020).

[10] Q. Li , Abyssal ocean overturning slowdown and warming driven by Antarctic meltwater. Nature **615**, 841–847 (2023). 36991191 10.1038/s41586-023-05762-w

[11] S. Zhou , Slowdown of Antarctic Bottom Water export driven by climatic wind and sea-ice changes. Nat. Clim. Change **13**, 701–709 (2023).

[12] M. R. Mazloff, P. Heimbach, C. Wunsch, An eddy-permitting Southern Ocean state estimate. J. Phys. Oceanogr. **40**, 880–899 (2010).

[13] J. S. Budge, D. G. Long, A comprehensive database for Antarctic iceberg tracking using scatterometer data. *IEEE J. Sel. Top. Appl. Earth Observ. Remote Sens*. **11**, 434–442 (2018).

[14] F. Fetterer, K. Knowles, W. N. Meier, M. Savoie, A. K. Windnagel, Sea Ice Index. (G02135, Version 3, 2017). [Data Set]. Boulder, Colorado USA. National Snow and Ice Data Center. 10.7265/N5K072F8.

[15] Y. Xie, V. Tamsitt, L. T. Bach, Localizing the Southern Ocean biogeochemical divide. Geophys. Res. Lett. **49**, e2022GL098260 (2022).

[16] A. H. Orsi, T. Whitworth, W. D. Nowlin, On the meridional extent and fronts of the Antarctic circumpolar current. Deep Sea Res. Part I Oceanogr. Res. Pap. **42**, 641–673 (1995).

[17] A. S. Studer , Antarctic Zone nutrient conditions during the last two glacial cycles. Paleoceanography **30**, 845–862 (2015).

[18] X. E. Ai , Southern Ocean upwelling, Earth’s obliquity, and glacial-interglacial atmospheric CO_2_ change. Science **370**, 1348–1352 (2020). 33303618 10.1126/science.abd2115

[19] F. Lambert , Dust-climate couplings over the past 800, 000 years from the EPICA Dome C ice core. Nature **452**, 616–619 (2008). 18385736 10.1038/nature06763

[20] F. Parrenin , Synchronous change of atmospheric CO_2_ and Antarctic temperature during the last deglacial warming. Science **339**, 1060–1063 (2013). 23449589 10.1126/science.1226368

[21] B. Bereiter , Revision of the EPICA Dome C CO_2_ record from 800 to 600 kyr before present. Geophys. Res. Lett. **42**, 542–549 (2015).

[22] C. E. Jasper , A 3.3-million-year record of Antarctic iceberg rafted debris and ice sheet evolution quantified by machine learning. Paleoceanogr. Paleoclimatol. **39**, e2024PA004897 (2024).

[23] M. E. Weber, N. R. Golledge, C. J. Fogwill, C. S. M. Turney, Z. A. Thomas, Decadal-scale onset and termination of Antarctic ice-mass loss during the last deglaciation. Nat. Commun. **12**, 6683 (2021). 34795275 10.1038/s41467-021-27053-6 PMC8602255

[24] M. E. Weber , Millennial-scale variability in Antarctic ice-sheet discharge during the last deglaciation. Nature **510**, 134–138 (2014). 24870232 10.1038/nature13397

[25] R. F. Anderson , Wind-driven upwelling in the Southern Ocean and the deglacial rise in atmospheric CO_2_. Science **323**, 1443–1448 (2009). 19286547 10.1126/science.1167441

[26] J. Gottschalk , Glacial heterogeneity in Southern Ocean carbon storage abated by fast South Indian deglacial carbon release. Nat. Commun. **11**, 6192 (2020). 33273459 10.1038/s41467-020-20034-1 PMC7712879

[27] T. Li , Enhanced subglacial discharge from Antarctica during meltwater pulse 1A. Nat. Commun. **14**, 7327 (2023). 37957152 10.1038/s41467-023-42974-0 PMC10643554

[28] J. E. Tierney , Glacial cooling and climate sensitivity revisited. Nature **584**, 569–573 (2020). 32848226 10.1038/s41586-020-2617-x

[29] K. E. Kohfeld, C. L. Quéré, S. P. Harrison, R. F. Anderson, Role of marine biology in glacial-interglacial CO_2_ cycles. Science **308**, 74–78 (2005). 15802597 10.1126/science.1105375

[30] S. L. Jaccard , Two modes of change in Southern Ocean productivity over the past million years. *Science* **339**, 1419–1423 (2013). 10.1126/science.1227545 23520109

[31] D. M. Sigman , The Southern Ocean during the ice ages: A review of the Antarctic surface isolation hypothesis, with comparison to the North Pacific. Quat. Sci. Rev. **254**, 106732 (2021).

[32] J. R. Toggweiler, J. L. Russell, S. R. Carson, Midlatitude westerlies, atmospheric CO_2_, and climate change during the ice ages. Paleoceanography **21**, PA2005 (2006).

[33] W. R. Gray , Poleward shift in the Southern Hemisphere westerly winds synchronous with the deglacial rise in CO2. Paleoceanogr. Paleoclimatol. **38**, e2023PA004666 (2023).

[34] R. Ferrari , Antarctic sea ice control on ocean circulation in present and glacial climates. Proc. Natl. Acad. Sci. U.S.A. **111**, 8753–8758 (2014). 24889624 10.1073/pnas.1323922111 PMC4066517

[35] A. J. Watson, G. K. Vallis, M. Nikurashin, Southern Ocean buoyancy forcing of ocean ventilation and glacial atmospheric CO2. Nat. Geosci. **8**, 861–864 (2015).

[36] D. M. Sigman, S. L. Jaccard, G. H. Haug, Polar Ocean stratification in a cold climate. Nature **428**, 59–63 (2004). 14999278 10.1038/nature02357

[37] A. Berger, M. F. Loutre, Insolation values for the climate of the last 10 million years. Quat. Sci. Rev. **10**, 297–317 (1991).

[38] R. Abernathey, D. Ferreira, Southern Ocean isopycnal mixing and ventilation changes driven by winds. Geophys. Res. Lett. **42**, 10357–10365 (2015).

[39] F. Fripiat ., Diatom bound nitrogen isotopes of sediment core PS69/899-2. PANGAEA. 10.1594/PANGAEA.983905. Deposited 7 January 2026.

[40] F. Fripiat ., Diatom bound nitrogen isotopes of sediment core PS69/899-2TC. PANGAEA. 10.1594/PANGAEA.983906. Deposited 7 January 2026.

[41] R. Gersonde, X. Crosta, A. Abelmann, L. Armand, Sea-surface temperature and sea ice distribution of the Southern Ocean at the EPILOG Last Glacial Maximum—A circum-Antarctic view based on siliceous microfossil records. Quat. Sci. Rev. **24**, 869–896 (2005).

[42] F. Fripiat *et al*., Age-depth model and stratigraphic tie points of sediment core PS69/899-2. PANGAEA. https://doi.org/10.1594/PANGAEA.983976. Deposited 7 January 2026.

[43] B. C. Lougheed, S. P. Obrochta, A. Rapid, Deterministic age-depth modeling routine for geological sequences with inherent depth uncertainty. Paleoceanogr. Paleoclimatol. **34**, 122–133 (2019).

[44] R. Hallberg, A. Gnanadesikan, The role of eddies in determining the structure and response of the wind-driven Southern Hemisphere overturning: Results from the Modeling Eddies in the Southern Ocean (MESO) Project. J. Phys. Oceanogr. **36**, 2232–2252 (2006).

[45] R. Abernathey, J. Marshall, D. Ferreira, The dependence of Southern Ocean meridional overturning on wind stress. J. Phys. Oceanogr. **41**, 2261–2278 (2011).

[46] A. K. Morrison, A. McC, Hogg, on the relationship between Southern Ocean overturning and ACC transport. J. Phys. Oceanogr. **43**, 140–148 (2013).

[47] E. C. Campbell , Antarctic offshore polynyas linked to Southern Hemisphere climate anomalies. Nature **570**, 319–325 (2019). 31182856 10.1038/s41586-019-1294-0

[48] L. Skinner , Rejuvenating the ocean: Mean ocean radiocarbon, CO_2_ release, and radiocarbon budget closure across the last deglaciation. Clim. Past **19**, 2177–2202 (2023).

[49] P. A. Rafter , Global reorganization of deep-sea circulation and carbon storage after the last ice age. Sci. Adv. **8**, eabq5434 (2022). 36383653 10.1126/sciadv.abq5434 PMC9668286

[50] A. Martínez-García , Iron fertilization of the subantarctic Ocean during the last ice age. Science **343**, 1347–1350 (2014). 24653031 10.1126/science.1246848

[51] F. Fripiat ., TEX86-based sea surface temperature reconstruction for sediment core PS69/899-2. PANGAEA. 10.1594/PANGAEA.983930. Deposited 7 January 2026.

[52] J. F. McManus, R. Francois, J.-M. Gherardi, L. D. Keigwin, S. Brown-Leger, Collapse and rapid resumption of Atlantic meridional circulation linked to deglacial climate changes. Nature **428**, 834–837 (2004). 15103371 10.1038/nature02494

[53] E. Böhm , Strong and deep Atlantic meridional overturning circulation during the last glacial cycle. Nature **517**, 73–76 (2015). 25517093 10.1038/nature14059

[54] M. J. Bentley , A community-based geological reconstruction of Antarctic Ice Sheet deglaciation since the Last Glacial Maximum. Quat. Sci. Rev. **100**, 1–9 (2014).

[55] N. R. Golledge , Antarctic contribution to meltwater pulse 1A from reduced Southern Ocean overturning. Nat. Commun. **5**, 5107 (2014). 25263015 10.1038/ncomms6107

[56] R. B. Alley, J. T. Andrews, D. C. Barber, P. U. Clark, Comment on “Catastrophic ice shelf breakup as the source of Heinrich event icebergs” by C. L. Hulbe et al. Paleoceanography **20**, 2004PA001086 (2005).

[57] M. Nikurashin, G. Vallis, A theory of deep stratification and overturning circulation in the ocean. *J. Phys. Oceanogr.* **41**, 485–502 (2011).

[58] A. Purich, M. H. England, Projected impacts of Antarctic meltwater anomalies over the twenty-first century. J. Climate **36**, 2703–2719 (2023).

[59] S. Schmidtko, K. J. Heywood, A. F. Thompson, S. Aoki, Multidecadal warming of Antarctic waters. Science **346**, 1227–1231 (2014). 25477461 10.1126/science.1256117

[60] C. A. Greene, D. D. Blankenship, D. E. Gwyther, A. Silvano, E. Wijk, Wind causes Totten Ice Shelf melt and acceleration. Sci. Adv. **3**, e1701681 (2017). 29109976 10.1126/sciadv.1701681 PMC5665591

[61] P. Spence , Localized rapid warming of West Antarctic subsurface waters by remote winds. Nat. Clim. Change **7**, 595–603 (2017).

[62] E. Rignot , Four decades of Antarctic Ice Sheet mass balance from 1979–2017. Proc. Natl. Acad. Sci. U.S.A. **116**, 1095–1103 (2019). 30642972 10.1073/pnas.1812883116 PMC6347714

[63] K. E. Kohfeld , Southern hemisphere westerly wind changes during the last glacial maximum: Paleo-data synthesis. Quat. Sci. Rev. **68**, 76–95 (2013).

[64] B. Bronselaer , Change in future climate due to Antarctic meltwater. Nature **564**, 53–58 (2018). 30455421 10.1038/s41586-018-0712-z

[65] L. Talley, Closure of the global overturning circulation through the Indian, Pacific, and Southern Oceans: Schematics and transports. Oceanography **26**, 80–97 (2013).

[66] G. H. Denton , The last glacial termination. Science **328**, 1652–1656 (2010). 20576882 10.1126/science.1184119

[67] M. P. Hain, D. M. Sigman, G. H. Haug, Distinct roles of the Southern Ocean and North Atlantic in the deglacial atmospheric radiocarbon decline. Earth Planet. Sci. Lett. **394**, 198–208 (2014).

[68] A. Gnanadesikan, A simple predictive model for the structure of the oceanic pycnocline. Science **283**, 2077–2079 (1999). 10092229 10.1126/science.283.5410.2077

[69] C. L. Stewart, P. Christoffersen, K. W. Nicholls, M. J. M. Williams, J. A. Dowdeswell, Basal melting of Ross Ice Shelf from solar heat absorption in an ice-front polynya. Nat. Geosci. **12**, 435–440 (2019).

[70] P. M. F. Sheehan, K. J. Heywood, Ross Ice Shelf frontal zone subjected to increasing melting by ocean surface waters. Science. Advances **10**, eado6429 (2024). 10.1126/sciadv.ado6429 PMC11546744 39514651

[71] E. L. Sikes, M. S. Cook, T. P. Guilderson, Reduced deep ocean ventilation in the Southern Pacific Ocean during the last glaciation persisted into the deglaciation. Earth Planet. Sci. Lett. **438**, 130–138 (2016).

[72] T. Chen , Synchronous centennial abrupt events in the ocean and atmosphere during the last deglaciation. Science **349**, 1537–1541 (2015). 26404835 10.1126/science.aac6159

[73] J. W. B. Rae , CO2 storage and release in the deep Southern Ocean on millennial to centennial timescales. Nature **562**, 569–573 (2018). 30356182 10.1038/s41586-018-0614-0

[74] M. Auger, R. Morrow, E. Kestenare, J.-B. Sallée, R. Cowley, Southern Ocean in-situ temperature trends over 25 years emerge from interannual variability. Nat. Commun. **12**, 514 (2021). 33479205 10.1038/s41467-020-20781-1 PMC7819991

[75] E. Ellison, A. Mashayek, M. Mazloff, The sensitivity of Southern Ocean air-sea carbon fluxes to background turbulent diapycnal mixing variability. JGR Oceans **128**, e2023JC019756 (2023).

[76] J. A. Baker , Continued Atlantic overturning circulation even under climate extremes. Nature **638**, 987–994 (2025). 40011721 10.1038/s41586-024-08544-0 PMC11864975

[77] B. Diekmann, Documentation of sediment core PS69/899-2. PANGAEA. (2007). 10.1594/PANGAEA.620965. Deposited 29 June 2007.

[78] D. M. Sigman , A bacterial method for the nitrogen isotopic analysis of nitrate in seawater and freshwater. Anal. Chem. **73**, 4145–4153 (2001). 11569803 10.1021/ac010088e

[79] M. A. Weigand, J. Foriel, B. Barnett, S. Oleynik, D. M. Sigman, Updates to instrumentation and protocols for isotopic analysis of nitrate by the denitrifier method. Rapid Commun. Mass Spectrom. **30**, 1365–1383 (2016). 27197029 10.1002/rcm.7570

[80] J. K. Böhlke, S. J. Mroczkowski, T. B. Coplen, Oxygen isotopes in nitrate: New reference materials for ^18^O: ^17^O: ^16^O measurements and observations on nitrate-water equilibration: Reference materials for O-isotopes in nitrate. Rapid Commun. Mass Spectrom. **17**, 1835–1846 (2003). 12876683 10.1002/rcm.1123

[81] F. Fripiat ., Diatom species composition of sediment core PS69/899-2. PANGAEA. 10.1594/PANGAEA.983907. Deposited 7 January 2026.

[82] R. Gersonde, U. Zielinski, The reconstruction of late quaternary Antarctic sea-ice distribution—the use of diatoms as a proxy for sea-ice. Palaeogeogr. Palaeoclimatol. Palaeoecol. **162**, 263–286 (2000).

[83] F. Fripiat ., Ice rafted debris count of sediment core PS69/899-2. PANGAEA. 10.1594/PANGAEA.983929. Deposited 7 January 2026.

[84] H. Grobe, A simple method for the determination of ice-rafted debris in sediment cores. Polarforschung **57**, 123–126 (1987).

[85] R. McKay , A comparison of methods for identifying and quantifying ice rafted debris on the Antarctic margin. Paleoceanogr. Paleoclimatol. **37**, e2021PA004404 (2022).

[86] J.-H. Kim , New indices and calibrations derived from the distribution of crenarchaeal isoprenoid tetraether lipids: Implications for past sea surface temperature reconstructions. Geochim. Cosmochim. Acta **74**, 4639–4654 (2010).

[87] S. Schouten, E. C. Hopmans, J. S. Sinninghe Damsté, The organic geochemistry of glycerol dialkyl glycerol tetraether lipids: A review. Org. Geochem. **54**, 19–61 (2013).

[88] F. Fripiat ., TEX86-based sea surface temperature reconstruction for sediment core PS69/899-2TC. PANGAEA. 10.1594/PANGAEA.983933. Deposited 7 January 2026.

[89] A. Auderset, M. Schmitt, A. Martínez-García, Simultaneous extraction and chromatographic separation of n-alkanes and alkenones from glycerol dialkyl glycerol tetraethers via selective accelerated solvent extraction. Org. Geochem. **143**, 103979 (2020).

[90] E. C. Hopmans, S. Schouten, J. S. Sinninghe Damsté, The effect of improved chromatography on GDGT-based palaeoproxies. Org. Geochem. **93**, 1–6 (2016).

[91] F. Fripiat ., Relative paleointensity (RPI) of the Earth’s magnetic field of sediment core PS69/899-2. PANGAEA. 10.1594/PANGAEA.983934. Deposited 7 January 2026.

[92] J. E. T. Channell, C. Xuan, D. A. Hodell, Stacking paleointensity and oxygen isotope data for the last 1.5 Myr (PISO-1500). Earth Planet. Sci. Lett. **283**, 14–23 (2009).

[93] L. Tauxe, Sedimentary records of relative paleointensity of the geomagnetic field: Theory and practice. Rev. Geophys. **31**, 319–354 (1993).

