# Supplementary material for: Deglacial stratification of the polar Southern Ocean
Source: Proc Natl Acad Sci U S A. 2026 Feb 2;123(6):e2502076123. doi: 10.1073/pnas.2502076123 (PMC12890832; doi:10.1073/pnas.2502076123)
Supplement: Supplementary file 1 — Appendix 01 (PDF) [file pnas.2502076123.sapp.pdf]

PNAS

Supporting Information for

Deglacial stratification of the polar Southern Ocean

François Fripiat[1,2,*], Daniel M. Sigman[3], Xuyuan E. Ai[2,3], Cédric Dumoulin[1], Simone Moretti[2], Anja S. Studer[4], Bernhard Diekmann[5], Oliver Esper[5], Thomas Frederichs[6], Frank Lamy[5], Ling Liu[5], Frank Pattyn[1], Mareike Schmitt[2], Ralf Tiedemann[5], Gerald H. Haug[2,7], and Alfredo Martínez-García[2]

François Fripiat
Email: francois.fripiat@ulb.be

This PDF file includes:

Supplementary text sections S1
Figures S1 to S9
Tables S1
SI References

Supplementary Information Text

S1 Sediment core chronology.

We determined the piston core's stratigraphy using a combination of stratigraphic markers (Table S1) and the *Undatable* age-depth modeling routine, which involves weighted random sampling from both age and depth uncertainties and a bootstrapping process across 10^5 simulations (1). *Undatable* generates median down-core ages, confidence intervals, and a probability density cloud representing all age-depth runs (Fig. S4). The following stratigraphic markers were used:

(i) The $\mathrm{TEX}_{86}^{\mathrm{L}}$-based sea surface temperature (SST) record from core PS69/899-2 was aligned with the Antarctic temperature stack (2) compiled from ice core data (Fig. S5a). We selected four $\mathrm{TEX}_{86}^{\mathrm{L}}$-based SST as stratigraphic tie-points (Table S1), marking the onset and end of the last two deglaciations, which are clearly defined in our SST records. An additional tie-point was assigned to the minimum temperature event during MIS5d. We applied an age uncertainty of ± 2 to ± 3 ka (95.4% CI) (represented by the gray shaded areas in Fig. S5a). Figure S6 illustrates the robustness of this approach by showing that shifting the $\mathrm{TEX}_{86}^{\mathrm{L}}$-based SST reconstruction forward or backward more than by 2 ka results in a loss of alignment. In addition, the direct comparison between $\mathrm{TEX}_{86}^{\mathrm{L}}$-based SST and diatom-bound δ^{15}N reconstructions *vs*. depth in the core PS69/899-2 show that transient deglacial δ^{15}N occurs during deglaciations, independently of the age-depth model (Fig. S7).

(ii) The relative paleointensity (RPI) record of core PS69/899-2 was aligned with the PISO-1500 paleointensity stack (Fig. S5b) (4, 5). Eleven stratigraphic tie-points were identified based on corresponding minima and maxima in both the PS69/899-2 RPI record and the PISO-1500 stack,

with age uncertainties ranging from ±2 ka to ±4 ka at a 95.4% confidence interval (gray areas in Fig. S5b). Most of these tie-points are also consistent with the SINT-800 paleointensity stack (6), particularly for the interval between 40 and 120 ka (Fig. S8). For tie-points younger than 40 ka, alignment with the paleointensity stacks is less pronounced; however, these tie-points align well with $\mathrm{TEX}_{86}^{\mathrm{L}}$-based SST and radiocarbon age constraints (Fig. S4; Table S1).

(iii) Three radiocarbon ages on total sedimentary organic matter (TOC) measured at the National Ocean Science Accelerator Mass Spectrometer facility in Woods Hole. Freeze-dried and homogenized samples were prepared for radiocarbon analysis using standard procedures (7). Radiocarbon ages have been calibrated using the Marine 20 calibration data (8) and by assuming a marine reservoir age of 1200 years for the Holocene and of 2200 years for glacial conditions (9, 10). Several complications may arise when using TOC instead of using biogenic carbonate for establishing ^{14}C chronology. Redistribution of organic matter including pre-aged material cannot be ruled out as a factor influencing the TOC age at the core site, yielding radiocarbon age older than stratigraphic ages. However, Zheng et al. (11) reported TOC ages in opal-rich Southern Ocean sediments systematically younger than the carbonate ages, likely from the adsorption of atmospheric CO_2 or volatile organic compounds to reactive opal surface sites. Notwithstanding these uncertainties, the two older radiocarbon ages are in good agreement with both SST and RPI-based stratigraphic tie points. To account for these uncertainties, we assigned relatively large uncertainties (± 2 to ± 3 ka, 95.4% CI) to these radiocarbon ages to estimate our age-depth model.

(iv) Last occurrence datum of the diatom *Rouxia leventerae* (130.0 ± 5.0 ka, 95.4% CI) (12).

We acknowledge considerable age uncertainties for much of the record (1.9 ± 0.7 ka, 68.2% CI). However, the deglaciation periods are more accurately constrained, aided by $\mathrm{TEX}_{86}^{\mathrm{L}}$-based stratigraphic tie-points (Figs. S4, S5, and S7), respectively. This is illustrated with a Pearson correlation coefficient of 0.71 (p-value of < 0.01) between TEX_{86}^{L}-based sea surface temperature (SST) record from core PS69/899-2 and the Antarctic temperature stack (2,3). The correlation is lower (Pearson correlation coefficient of 0.48), but still significant (p-value of < 0.01), between the RPI records for core PS69/899-2 and PISO-1500 paleointensity stack, but these stratigraphic tie-points are mostly used to constrain the age-depth relationship during glacial periods.

To determine the age of the bottom portion of the trigger core (PS69/899-2TC), we used $\mathrm{TEX}_{86}^{\mathrm{L}}$-derived sea surface temperatures and diatom-bound δ^{15}N to align it with the piston core, establishing an age of 8.0 ka. We then applied the linear accumulation rates from the piston core to the trigger core, resulting in an estimated age of 0.4 ka for the topmost 1 cm of the trigger core. While we recognize this approach has limitations, it primarily affects Holocene samples, which are not the focus of our study.

The age-depth model's down-core uncertainties were used to generate 68.2% confidence intervals for the diatom-bound δ^{15}N record (shown as red error bars in Figs. 2D, 4D,E, and 5K,L).

The age-depth model for core MD12-3394 – one of three APF diatom-bound δ^{15}N records (2) – was slightly adjusted for the last deglaciation by adding a single stratigraphic tie-point to improve the match with deglacial temperature trends, i.e., to better align the TEX86-based SST record with the Antarctic temperature stack (Fig. S9a). However, considering the associated uncertainties, we now use the average of the original and adjusted age-depth models in our analysis (Fig. S9b).

For the APF δ^{15}N stack (shown as black solid line in Figs. 4D,E and 5K,L), combining the PS75/072-4, MD11-3353, and MD12-3394 diatom-bound δ^{15}N records near the APF, we applied a Monte Carlo simulation and a Kalman filter on a 500-year time grid (2), generating five hundred synthetic time series by introducing errors to both the age (uncertainty of 1-2 ka) and diatom-bound δ^{15}N (uncertainty of 0.08 ‰). The APF δ^{15}N stack is the average of these five hundred synthetic time series.

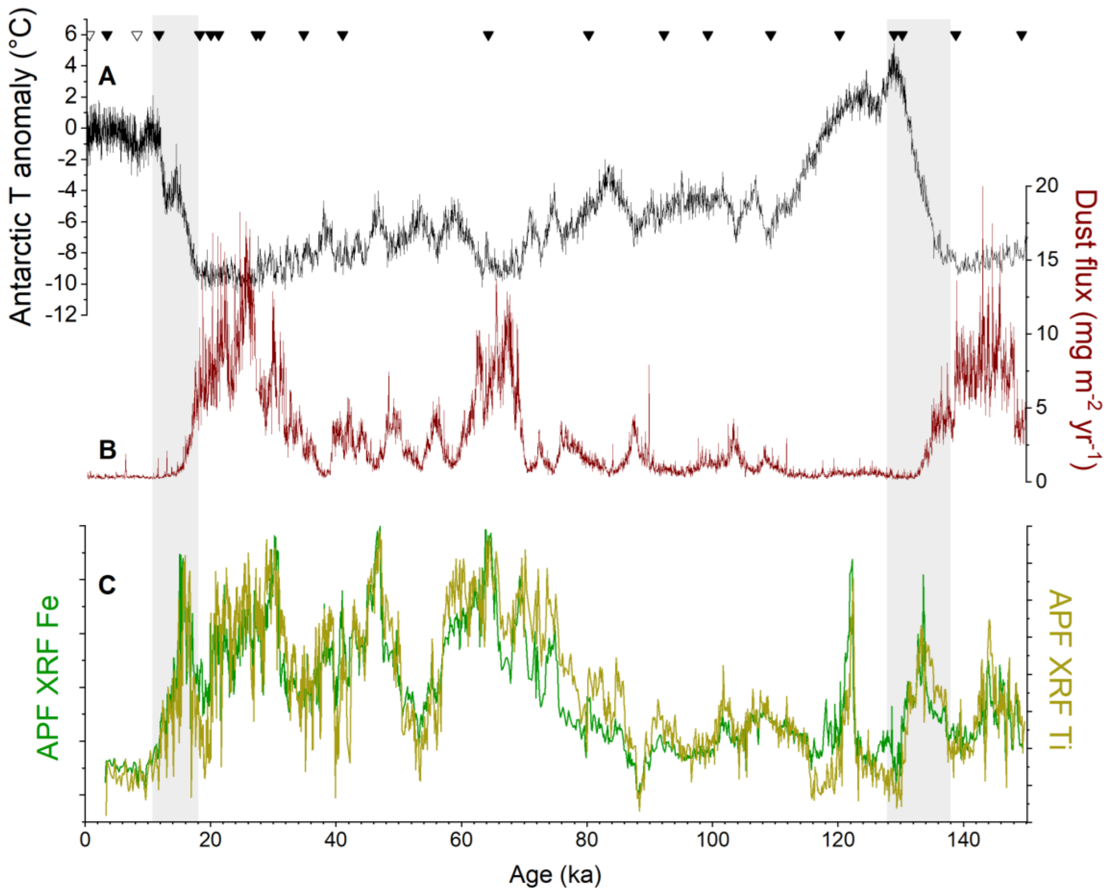

Fig. S1. (A) Reconstruction of air temperature anomaly in Antarctica (3). (B) Dust flux records in the EPICA Dome C (EDC) ice core (13). (C) XRF Ti and Fe concentrations of PS69/899-2. Gray shaded areas indicate the deglaciations. The age model tie-points for PS69/899-2 are indicated with triangles along the top (filled symbols for the piston core and open symbols for the trigger core).

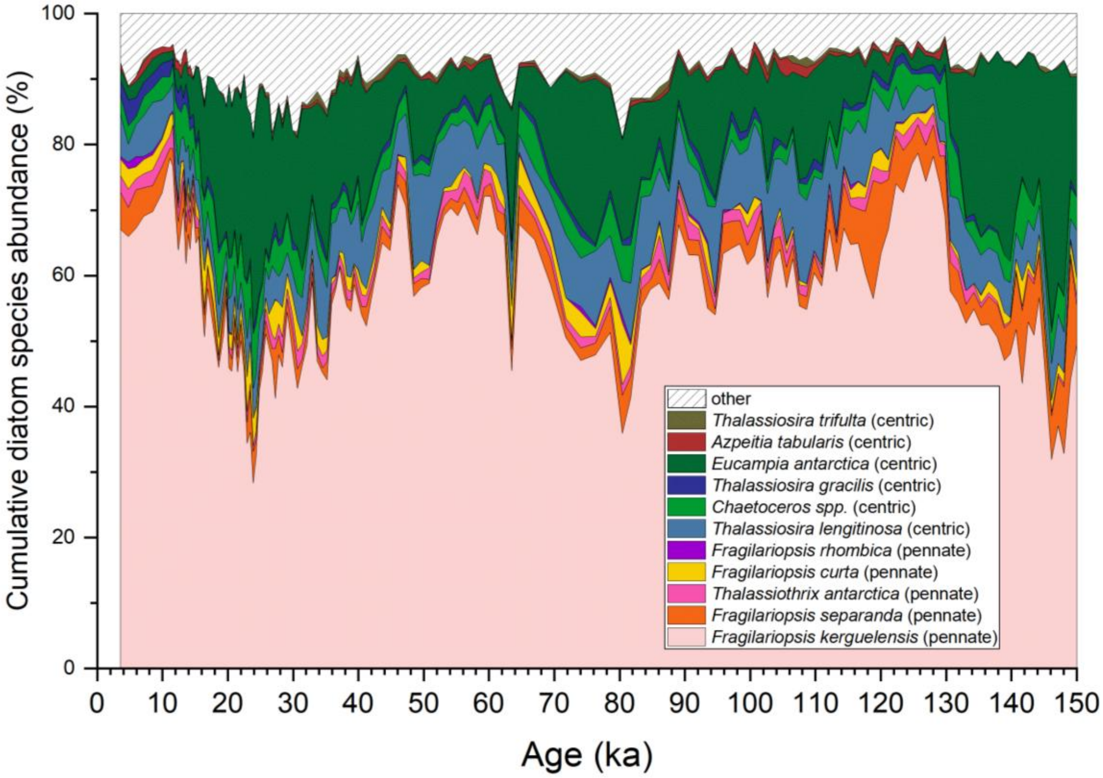

Fig. S2. Diatom species counts in sediment core PS69/899-2. The diatom species are grouped into pennates and centrics and arranged from bottom up in order of decreasing abundance. Reported are the 11 most abundant diatom species present in core PS69/899-2, representing more than 85% of the diatom assemblage. Pennate diatoms are pink-purple colored, centric diatoms blue-green. *Fragilariopsis kerguelensis* dominates the total diatom assemblage (30-80%), followed by *Eucampia antarctica* (10-30%).

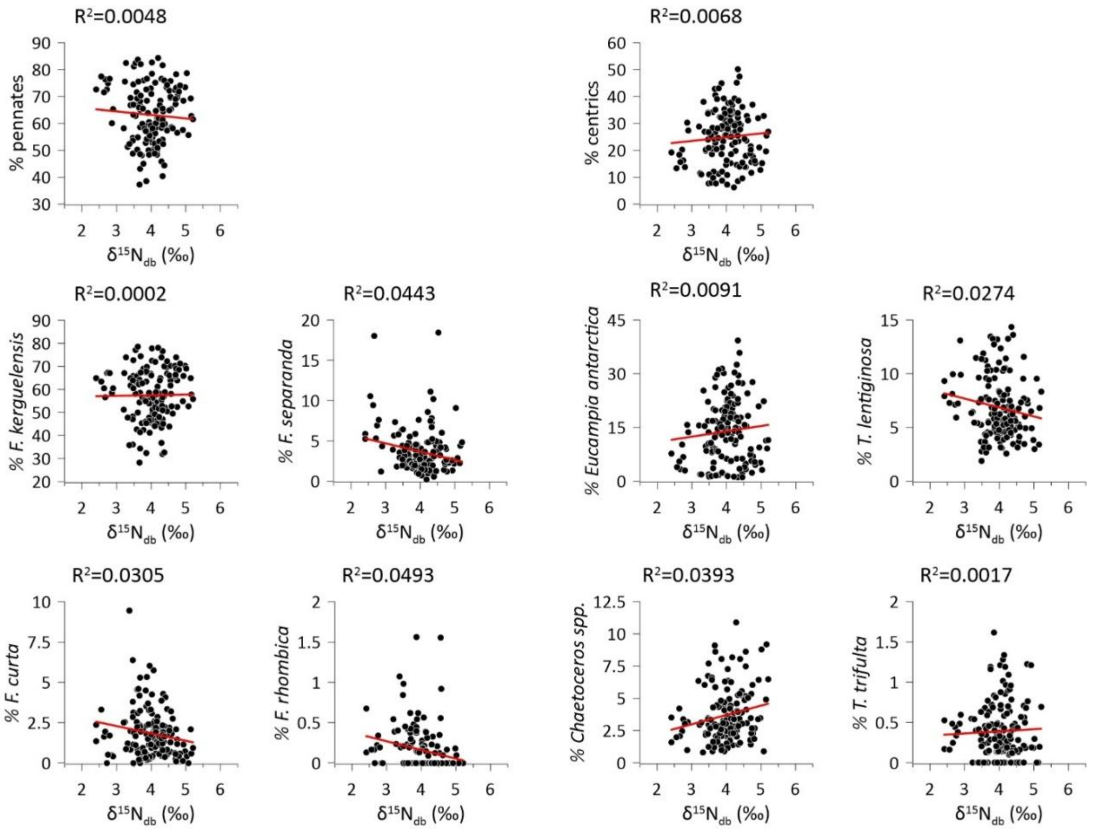

Fig. S3. Correlation of diatom assemblages and species abundance to diatom-bound $\delta^{15}N$ values for core PS69/899-2. The diatom species are grouped into pennates (left) and centrics (right) and arranged in order of decreasing abundance.

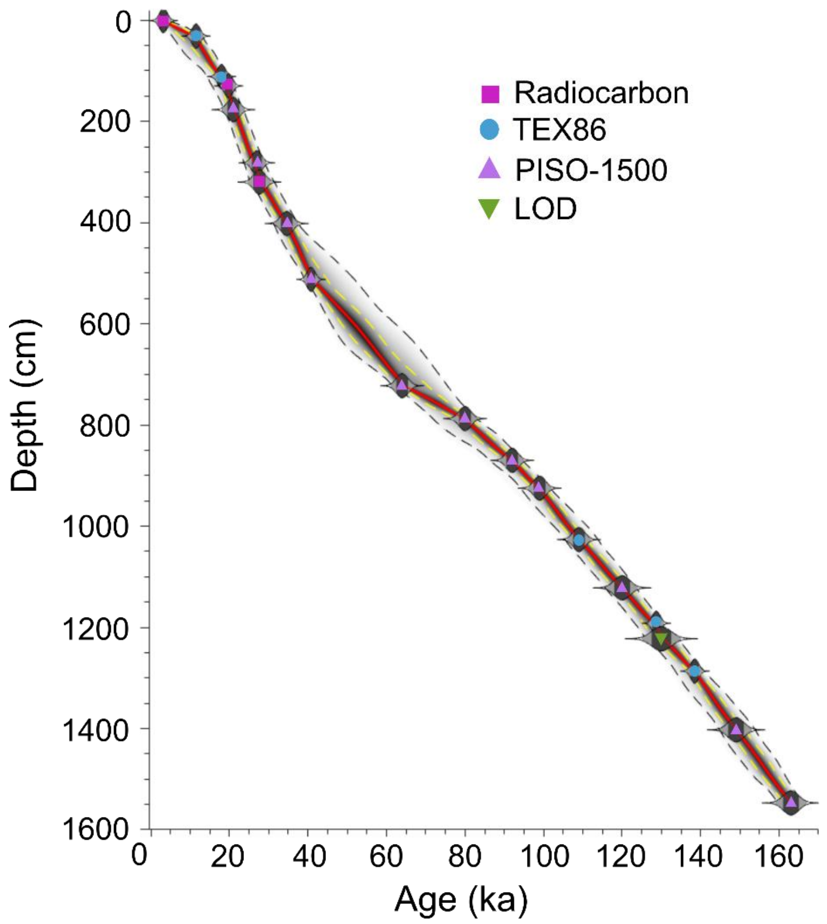

Fig. S4. Age-depth model for the PS69/899-2 core based on the last occurrence of the diatoms *Rouxia leventerae* (green triangle), cross-correlation between the organic biomarker $\mathbf{TEX_{86}^{L}}$-based sea-surface temperature and the Antarctic temperature stack (light blue circles) (Fig. S5a) (2, 3), cross-correlation between the relative paleointensity (RPI) of core PS69/899-2 and the PISO-1500 paleointensity stack (purple triangles) (Fig. S5b) (4, 5), and the AMS radiocarbon dates on total sedimentary organic matter (pink squares). The age-depth model has been generated using the *Undatable* age-depth modelling routine (1). The red line is the median, and the yellow and black dashed lines represent the 68.2% and 95.4% confidence intervals, respectively.

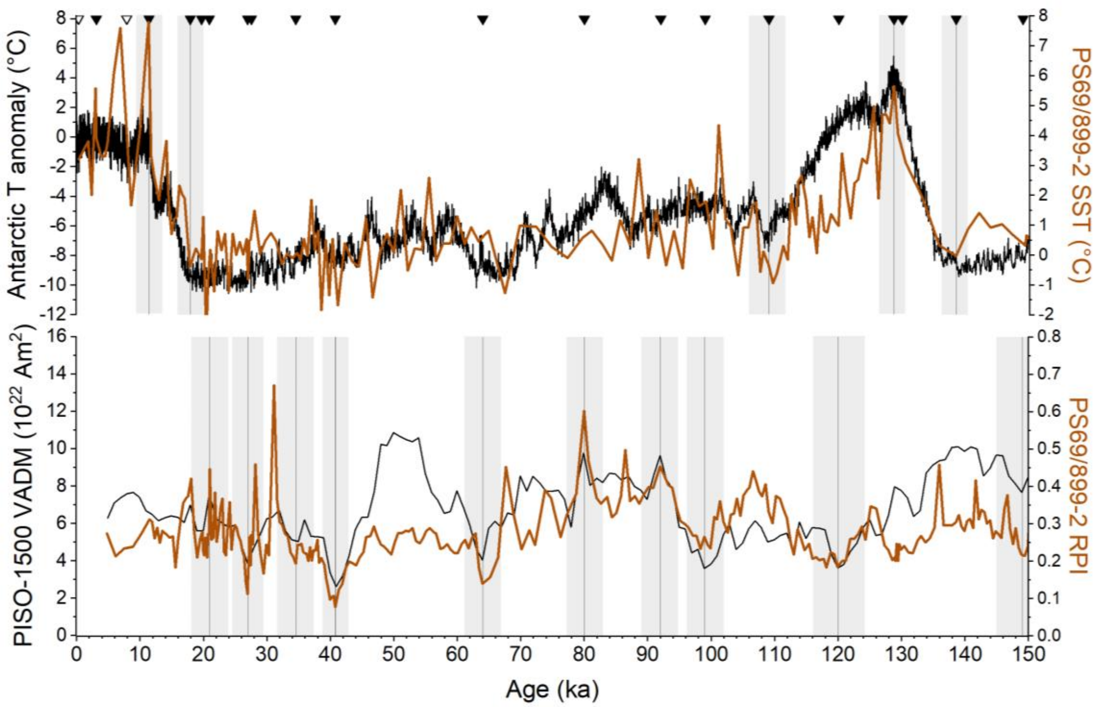

Figure S5. (A) $\mathrm{TEX}_{86}^{\mathrm{L}}$-based sea-surface temperature (SST) of PS69/899-2 (brown) compared with the reconstruction of air temperature anomaly in Antarctica (black) (2, 3). (B) Relative paleointensity (RPI) for core PS69/899-2 (brown) compared with the virtual axial dipole movement (VADM) calibration of the PISO-1500 relative paleointensity stack (4, 5) (black). Vertical lines mark SST and RPI tie-points used from this cross-correlation analysis for the age model shown in Fig. S4. The gray areas represent the assigned 95.4% confidence intervals to the tie-points. The age model tie-points for PS69/899-2 are indicated with triangles along the top (filled symbols for the piston core and open symbols for the trigger core).

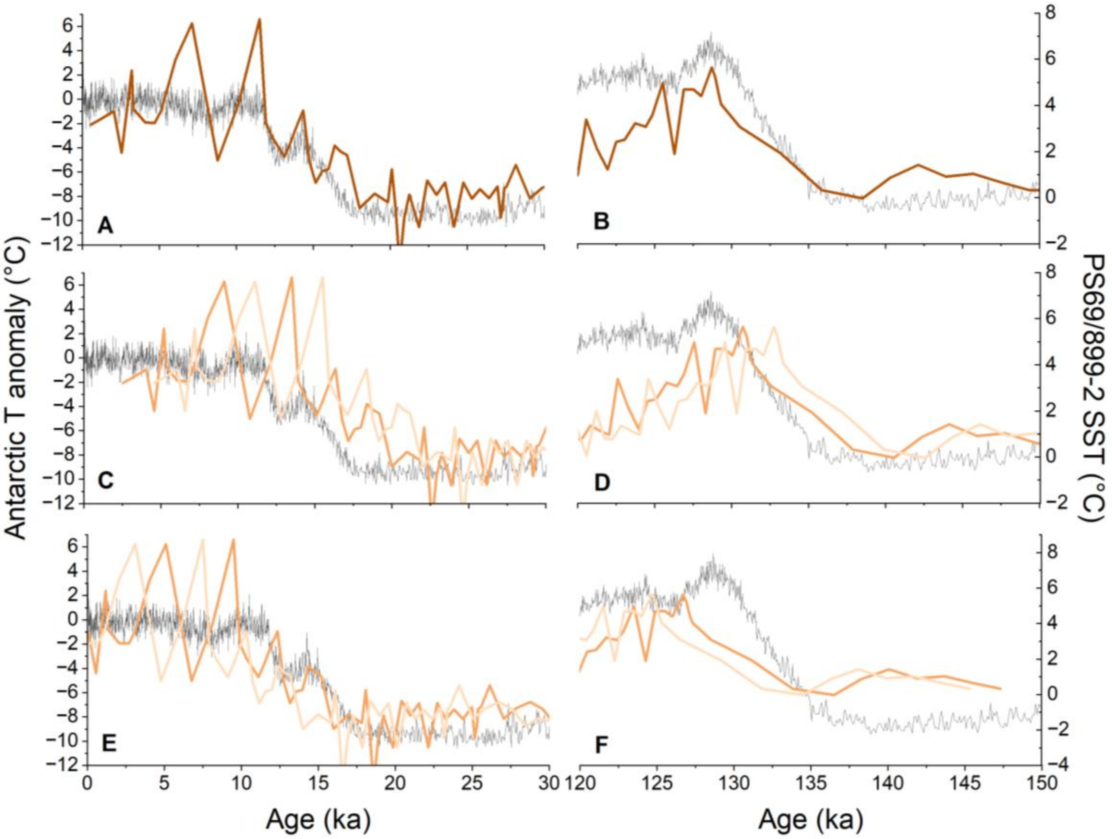

Fig. S6. $\mathbf{TEX_{86}^{L}}$-based sea-surface temperature (SST) of PS69/899-2 (brown) compared with the reconstruction of air temperature anomaly in Antarctica (black) **(2, 3)**, for the last (A) and the penultimate (B) deglaciation. (C,D) Same as (A,B) but with the age-depth model shifted by 2 and 4 ka backward, represented by increasingly lighter shades of brown. (E,F) Same as (A,B) but with the age-depth model shifted by 2 and 4 ka backward, represented by increasingly lighter shades of brown.

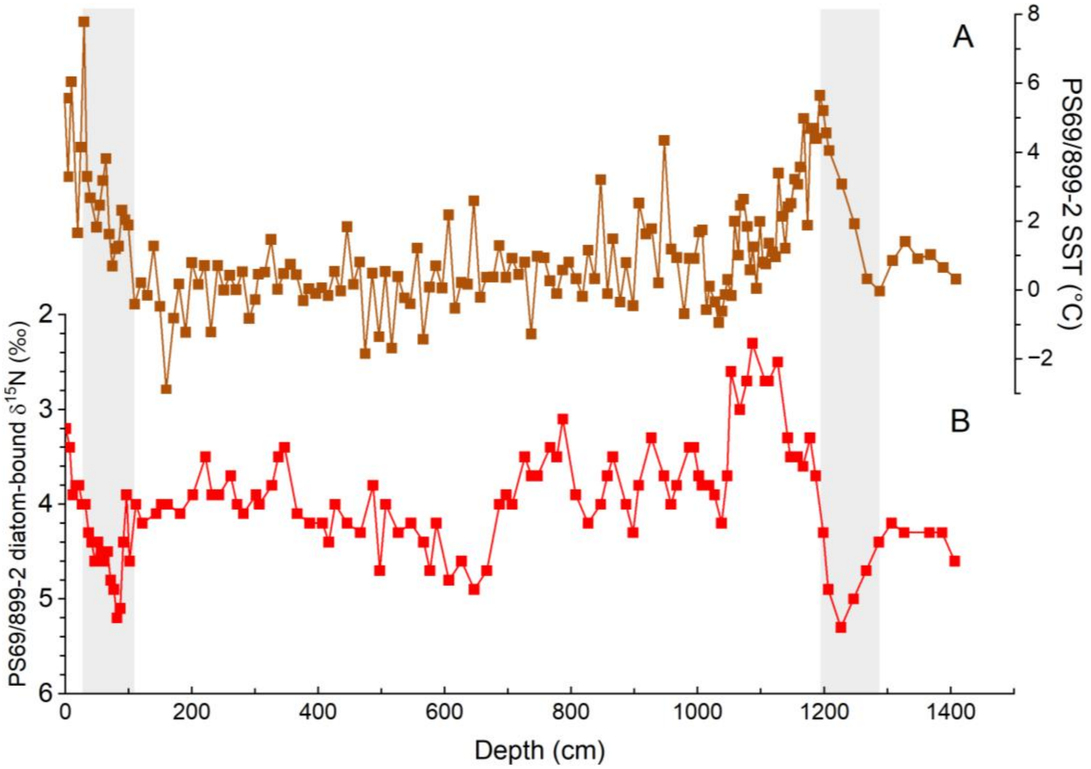

Figure S7: (A) $\mathbf{TEX}_{86}^{L}$-based sea-surface temperature (SST) and (B) diatom-bound $\delta^{15}N$ vs depth in PS69/899-2 core.

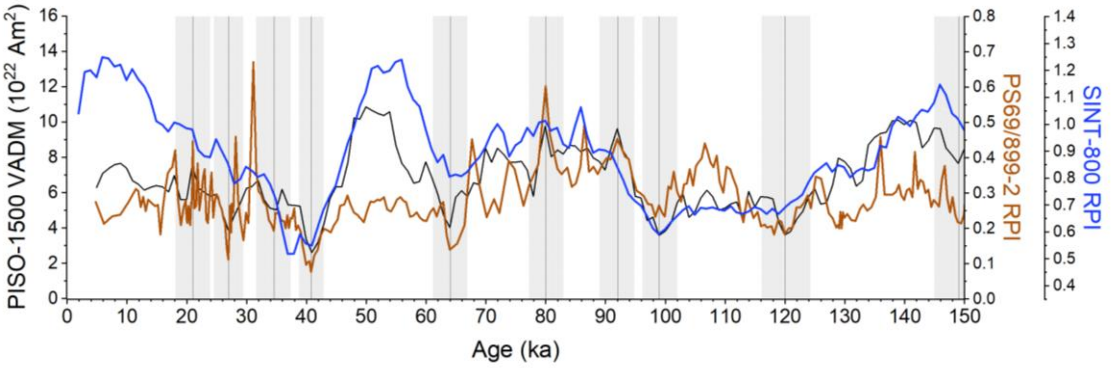

Figure S8. Relative paleointensity (RPI) for core PS69/899-2 (brown) compared with the virtual axial dipole movement (VADM) calibration of the PISO-1500 relative paleointensity stack (4, 5) (black) and the SINT-800 relative paleointensity stack (6) (blue). Vertical lines mark RPI tie-points used from this cross-correlation analysis for the age model shown in Fig. S4. The gray areas represent the assigned 95.4% confidence intervals to the tie-points.

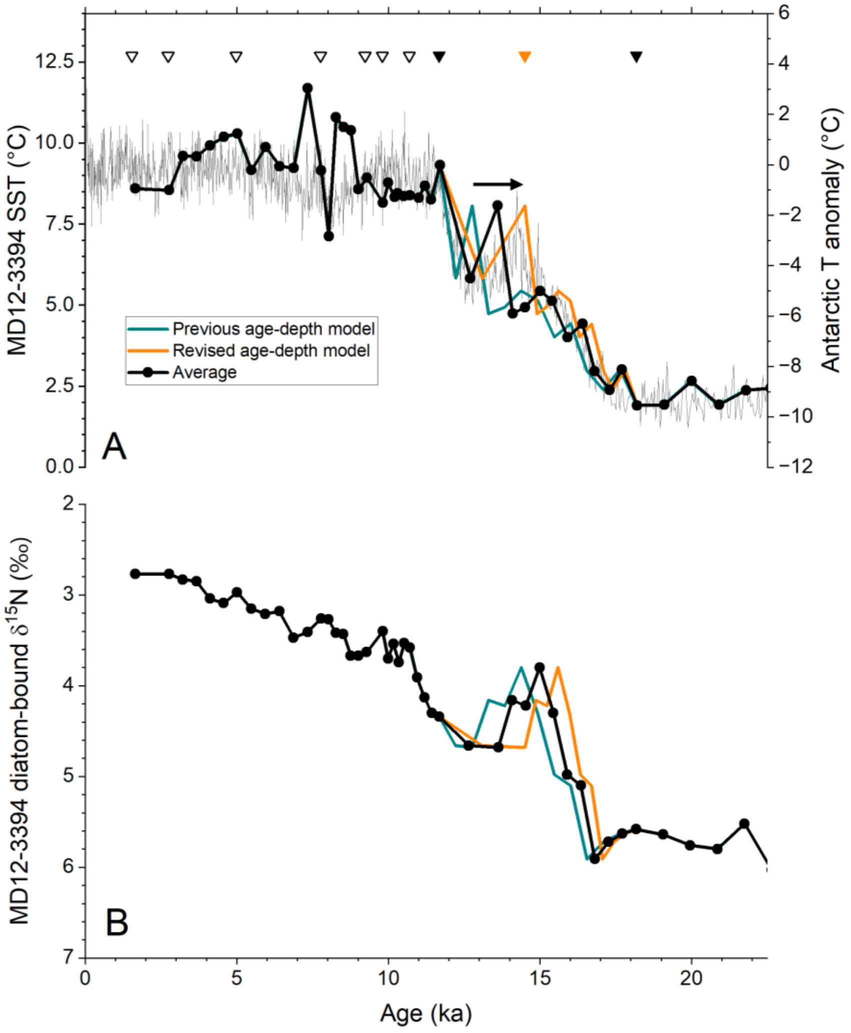

Figure S9. (A) TEX_{86}^{L}-based sea-surface temperature (SST) of core MD12-3394 compared with the reconstruction of air temperature anomaly in Antarctica (black) (2, 3). Green line is the published age-depth model for MD12-3394 (2), and orange line is an adjusted age-depth model for the last deglaciation based on one additional tie-point (orange symbol in (A)) to better align the sharpest deglacial TEX_{86}^{L}-based SST increase with the fastest warming Antarctic air temperature. The black line with circles is the average age-depth model that we use in this study. (B) Diatom-bound $\delta^{15}N$ of core MD12-3394 using the three age-depth models described in (A). The age model tie-points for MD12-3394 are indicated with triangles along the top (filled symbols for radiocarbon ages, open symbols for TEX_{86}^{L}-based SST tie-points, and orange symbol for the additional TEX_{86}^{L}-based SST tie-point during deglaciation).

Stratigraphic markers	upper depth	lower depth	age	95.4% CI
	(cm)	(cm)	(Ka)	(Ka)
bulk radiocarbon age	0.0	2.0	3.2	1.2 – 5.2
TEX_{86}^{L}-SST vs. Ant. T°C stack	30.5	32.5	11.5	9.5 – 13.5
TEX_{86}^{L}-SST vs. Ant. T°C stack	110.5	112.5	18.0	16.0 - 20.0
bulk radiocarbon age	129.0	131.0	19.8	17.8 – 21.8
RPI vs. PISO-1500 stack	176.5	177.5	21.0	18.0 – 24.0
RPI vs. PISO-1500 stack	281.5	282.5	27.0	24.5 – 29.5
bulk radiocarbon age	319.0	321.0	27.6	25.6 – 29.6
RPI vs. PISO-1500 stack	401.5	402.5	34.6	31.6 – 37.6
RPI vs. PISO-1500 stack	512.0	513.0	40.8	38.8 – 42.8
RPI vs. PISO-1500 stack	722.0	723.0	64.0	61.0-67.0
RPI vs. PISO-1500 stack	787.0	788.0	80.0	77.7 – 83.0
RPI vs. PISO-1500 stack	869.5	870.5	92.0	89.0 – 95.0
RPI vs. PISO-1500 stack	924.5	925.5	99.0	96.0 - 102.0
TEX_{86}^{L}-SST vs. Ant. T°C stack	1025.5	1027.5	109.0	106.0 - 112.0
RPI vs. PISO-1500 stack	1122.0	1122.5	120.0	116.0-124.0
TEX_{86}^{L}-SST vs. Ant. T°C stack	1191.0	1193.0	128.7	126.7 – 130.7
LOD *Rouxia leventerae*	1222.0	1223.0	130.0	125.0 - 135.0
TEX_{86}^{L}-SST vs. Ant. T°C stack	1285.50	1287.5	138.5	136.5 – 140.5
RPI vs. PISO-1500 stack	1402.5	1403.5	149.0	145.0 – 153.5
RPI vs. PISO-1500 stack	1546.5	1547.5	163.0	159.0-167.0

Table S1. Age model tie-points for PS69/899-2 for the age-depth model shown in Fig. S4.

SI References

1. B. C. Lougheed, S. P. Obrochta, A Rapid, Deterministic Age-Depth Modeling Routine for Geological Sequences With Inherent Depth Uncertainty. *Paleoceanography and Paleoclimatology* **34**, 122–133 (2019).
2. X. E. Ai, *et al.*, Southern Ocean upwelling, Earth's obliquity, and glacial-interglacial atmospheric CO_2 change. *Science* **370**, 1348–1352 (2020).
3. F. Parrenin, *et al.*, Synchronous Change of Atmospheric CO_2 and Antarctic Temperature During the Last Deglacial Warming. *Science* **339**, 1060–1063 (2013).
4. J. E. T. Channell, C. Xuan, D. A. Hodell, Stacking paleointensity and oxygen isotope data for the last 1.5 Myr (PISO-1500). *Earth and Planetary Science Letters* **283**, 14–23 (2009).
5. J. E. T. Channell, B. S. Singer, B. R. Jicha, Timing of Quaternary geomagnetic reversals and excursions in volcanic and sedimentary archives. *Quaternary Science Reviews* **228**, 106114 (2020).
6. Y. Guyodo, J.-P. Valet, Global changes in intensity of the Earth's magnetic field during the past 800 kyr. *Nature* **399**, 249–252 (1999).
7. A. P. McNichol, E. A. Osborne, A. R. Gagnon, B. Fry, G. A. Jones, TIC, TOC, DIC, DOC, PIC, POC — unique aspects in the preparation of oceanographic samples for 14C-AMS. *Nuclear Instruments and Methods in Physics Research Section B: Beam Interactions with Materials and Atoms* **92**, 162–165 (1994).
8. T. J. Heaton, *et al.*, Marine20—The Marine Radiocarbon Age Calibration Curve (0–55,000 cal BP). *Radiocarbon* **62**, 779–820 (2020).
9. P. A. Rafter, *et al.*, Global reorganization of deep-sea circulation and carbon storage after the last ice age. *Science Advances* **8**, eabq5434 (2022).
10. L. Skinner, *et al.*, Rejuvenating the ocean: mean ocean radiocarbon, CO_2 release, and radiocarbon budget closure across the last deglaciation. *Climate of the Past* **19**, 2177–2202 (2023).
11. Y. Zheng, *et al.*, Challenges in Radiocarbon Dating Organic Carbon in Opal-Rich Marine Sediments. *Radiocarbon* **44**, 123–136 (2002).
12. U. Zielinski, R. Gersonde, Plio–Pleistocene diatom biostratigraphy from ODP Leg 177, Atlantic sector of the Southern Ocean. *Marine Micropaleontology* **45**, 225–268 (2002).
13. F. Lambert, *et al.*, Dust-climate couplings over the past 800,000 years from the EPICA Dome C ice core. *Nature* **452**, 616–619 (2008).
